# Zipfian Distributions in Child-Directed Speech

**DOI:** 10.1162/opmi_a_00070

**Published:** 2023-01-24

**Authors:** Ori Lavi-Rotbain, Inbal Arnon

**Affiliations:** The Edmond and Lilly Safra Center for Brain Sciences, Hebrew University, Jerusalem, Israel; Department of Psychology, Hebrew University, Jerusalem, Israel

**Keywords:** Child-Directed Speech, Zipfian distribution, language learning

## Abstract

Across languages, word frequency and rank follow a power law relation, forming a distribution known as the Zipfian distribution. There is growing experimental evidence that this well-studied phenomenon may be beneficial for language learning. However, most investigations of word distributions in natural language have focused on adult-to-adult speech: Zipf’s law has not been thoroughly evaluated in child-directed speech (CDS) across languages. If Zipfian distributions facilitate learning, they should also be found in CDS. At the same time, several unique properties of CDS may result in a less skewed distribution. Here, we examine the frequency distribution of words in CDS in three studies. We first show that CDS is Zipfian across 15 languages from seven language families. We then show that CDS is Zipfian from early on (six-months) and across development for five languages with sufficient longitudinal data. Finally, we show that the distribution holds across different parts of speech: Nouns, verbs, adjectives and prepositions follow a Zipfian distribution. Together, the results show that the input children hear is skewed in a particular way from early on, providing necessary (but not sufficient) support for the postulated learning advantage of such skew. They highlight the need to study skewed learning environments experimentally.

## INTRODUCTION

**Figure 1.**
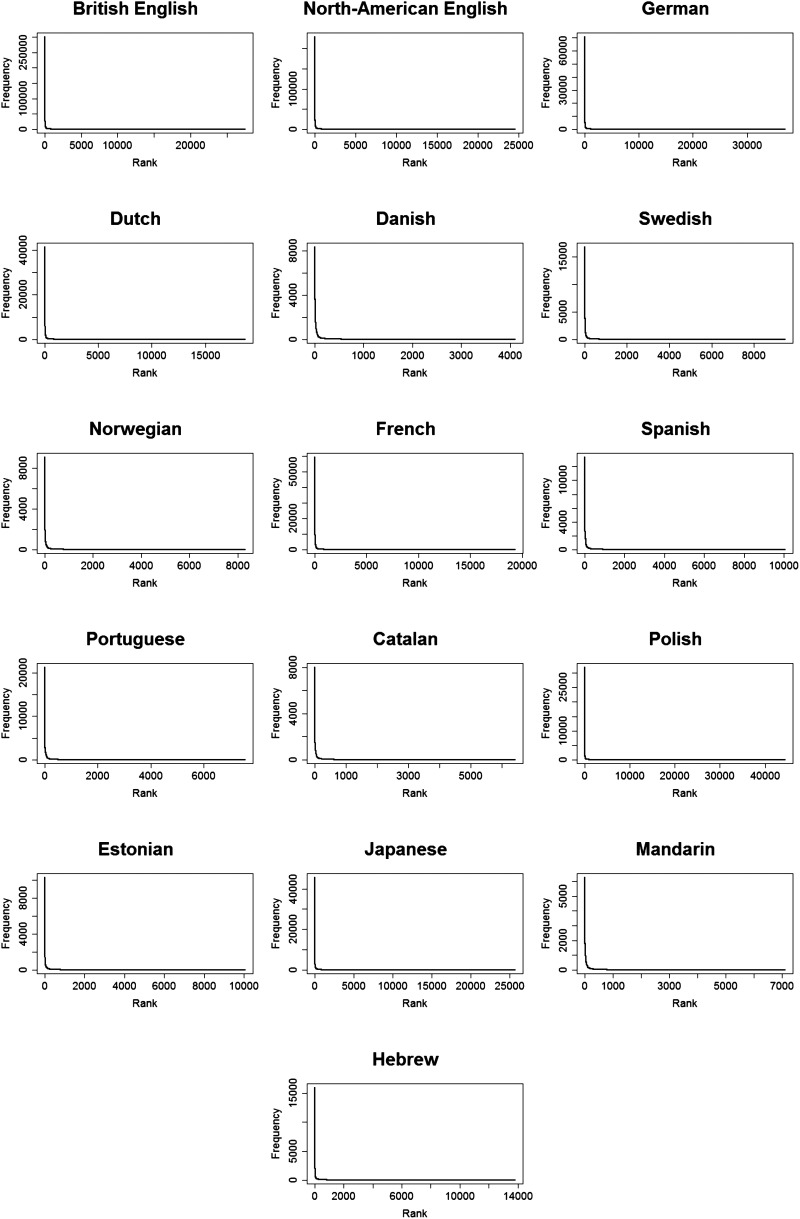
The observed word distributions for each language.

**Figure 2.**
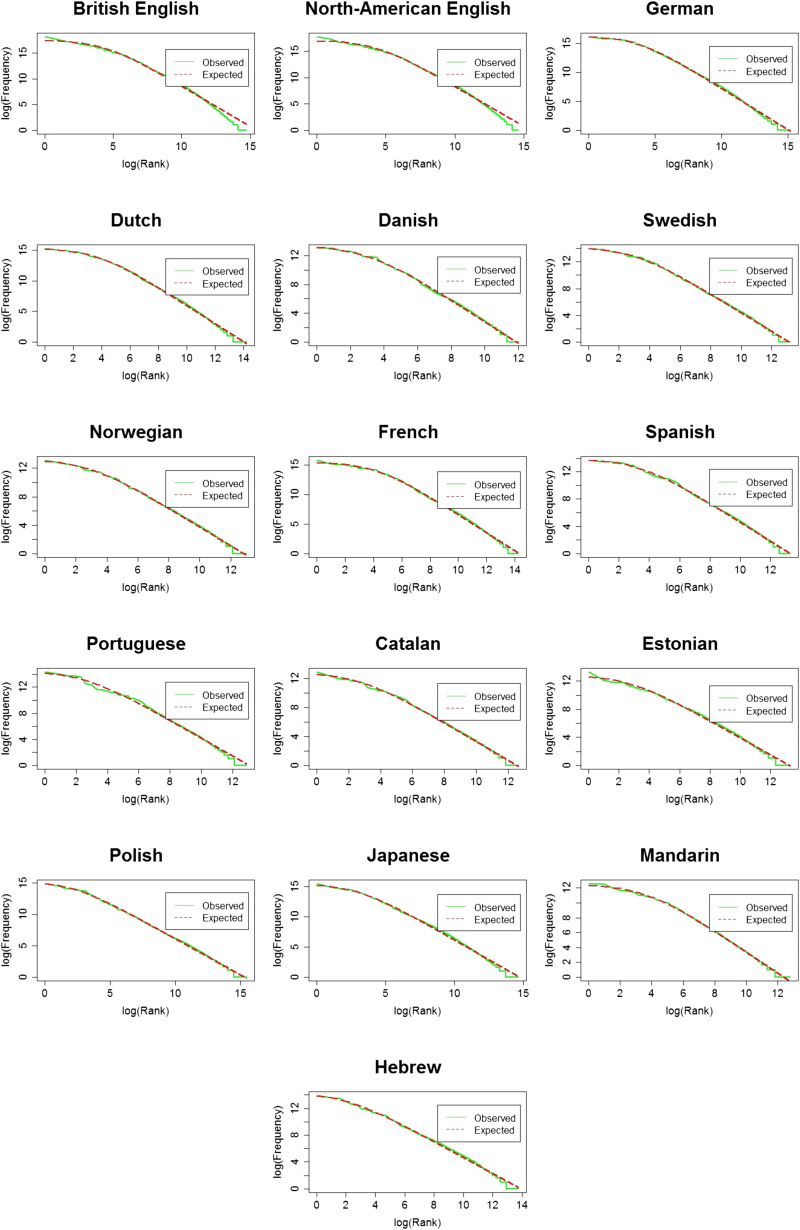
The observed (in green) and expected (in red) word distributions for each language on a log-log scale.

When it comes to frequency, not all words are created equal: it is known that words in language are not uniformly distributed. Instead, a small number of word types accounts for most of the tokens across languages. In English for example, 0.3% of word types account for 50% of all tokens, with the remaining words having very low frequencies (Yang, 2013). The relation between a word’s rank and its frequency can be described using a power law: Differences in frequency are large among the most frequent words, and almost non-existent among the least frequent words. This power law relation results in a highly skewed frequency distribution with a narrowed peak for the most frequent words, and a very long tail for the low frequency words (Figure 1). This distribution is known as the Zipfian distribution (Zipf, 1949), and is characterized by a linear relation between log frequency and log rank (Figure 2). Natural language does not follow Zipf’s law completely, with consistent prediction errors on both edges of the frequency scale (Montemurro, 2001; Piantadosi, 2014). For example, the most frequent words are not as frequent as they are expected to be. We follow Piantadosi (2014) and use the term “near-Zipfian” to refer to a distribution where Zipf’s law holds approximately.

Near-Zipfian distributions are found across languages, for multiple linguistic domains: They are found when looking at the entire lexicon, at different parts of speech separately, and even for specific semantic classes such as taboo words (Bentz, Alikaniotis, Samardžić, & Buttery, 2017; Ferrer-i-Cancho, 2005; Mehri & Jamaati, 2017; Piantadosi, 2014). There are many different explanations for the origin of Zipfian distributions in language, with ongoing controversy about the significance of this law and whether it tells us something fundamental about language or not (e.g., Ferrer-i-Cancho et al., 2020). On the one hand, such distributions are found in numerous domains across the physical world - from the distribution of visual objects (Clerkin et al., 2017) and their co-occurrences (Lavi-Rotbain & Arnon, 2021), to population size in American cities (Clauset et al., 2009) and crater size on the moon (see Newman, 2005 for a review) - where they are thought to reflect general mathematical principles not unique to language (e.g., scale-invariance: Chater & Brown, 1999). On the other hand, language, unlike the physical world, is created and shaped by humans (Ferrer-i-Cancho et al., 2020; Piantadosi, 2014; Semple et al., 2022). Consequently, the recurrence and preservation of Zipfian distributions in language may reflect foundational properties of human language or cognition (Christiansen & Chater, 2008; Ferrer-i-Cancho et al., 2020; Gibson et al., 2019; Piantadosi, 2014; Semple et al., 2022).

Some explanations emphasize the impact of language being a communication system under cognitive pressures, and suggest that Zipfian distributions emerge spontaneously under the pressure to minimize listener and speaker efforts (Ferrer-i-Cancho & Sole, 2003), or from the need for easy and fast communication (Ferrer-i-Cancho, 2016). A similar perspective emphasizes the role of Shannon’s information theory (Shannon, 1948): Given that language is a noisy communication channel, there is pressure for optimal coding of the lexicon (Coupé et al., 2019; Ferrer-i-Cancho et al., 2020). Zipfian distributions are seen as a form of optimal coding under such accounts. These explanations attribute the presence of Zipfian distributions to the communicative and cognitive pressures that impact language learning and use.

A different line of work highlights semantics as the driving force of Zipfian distributions. Manin (2008) suggests a mechanism by which the tendency to avoid excessive synonymy results in a Zipfian distribution. According to his model, the measure of specificity of a word is closely related to frequency: Words that are more generic tend to be more frequent (e.g. “city”), while words that are more specific are less frequent (e.g., “London”). The variance in specificity level across words, together with the changes in words’ meaning over time and with the pressure to avoid excessive synonymy (using a word that is too specific for a certain scenario), lead to a Zipfian distribution in which the semantic space can be divided to layers of varying specificity (Manin, 2008). In a more recent model, Lestrade (2017) adds to Manin’s specificity model and claims that semantics alone is not enough: it takes both semantics and syntax in order to form a Zipfian distribution. According to Lestrade, syntax leads to division of words into parts-of-speech categories that differ greatly in size. As in Manin (2008), Lestrade claims that semantics leads to a variation in specificity level across words from the same part-of-speech category. Using a computational model, Lestrade shows that only when accounting for both semantics and syntax, the resulted distribution is Zipfian (Lestrade, 2017).

A host of additional explanations tie the presence of Zipfian distributions in language to learnability pressures (Bentz, Alikaniotis, Cysouw, & Ferrer-i-Cancho, 2017; Coupé et al., 2019; Lavi-Rotbain & Arnon, 2019, 2020, 2022). Skewed distributions, like the Zipfian one, are more predictable than uniform distributions, making it easier to guess the next word. This increased predictability may confer a learnability advantage for certain aspects of language learning, like segmenting and learning words, for several reasons. First, when it is easier to predict upcoming elements, processing resources can be used more efficiently. In addition, high frequency words can be learned early on, and used to facilitate learning of lower frequency elements, as can be seen in infants use of their own name to segment adjacent words (Bortfeld et al., 2005), or in the use of familiar words to segment novel phonologically similar words (Altvater-Mackensen & Mani, 2013). Finally, lower frequency words may also benefit from appearing next to high frequency ones: the contrast between high and low frequency could make the lower frequency words easier to identify or learn. The facilitative potential of the contrast between high and low frequency is supported by findings from several domains. In a phenomenon known as stimulus-specific adaptation, rare tones cause an increased neuronal reaction in rats, but only when they were rare enough (Rubin et al., 2016). Similarly, in memory tasks, adults show improved memory for novel items only when these items were relatively rare (Reggev et al., 2018).

The idea that Zipfian distributions may facilitate learning is starting to gain empirical support. The vast majority of lab-based studies looking at word segmentation and learning use a uniform distribution where each item appears the same number of times (e.g., Saffran et al., 1996). This allows researchers to better control for frequency effects, but does not reflect the natural skew of words in language. However, several recent studies compared learning in Zipfian and uniform distributions and found facilitative effects: Across different aspects of language learning, including mapping between phrasal form and meaning, word segmentation, noun learning, and category formation, exposure to Zipfian distributions did not harm learning, despite the lower frequency of certain elements (Kurumada et al., 2013; Schuler et al., 2017) and even led to facilitation in certain cases (Goldberg et al., 2004; Hendrickson & Perfors, 2019; Lavi-Rotbain & Arnon, 2019, 2020, 2022). Importantly, if the propensity of this distribution in language can be explained (even partially) by its learning advantage, then language directed to learners should also be Zipfian. If it is not, then the facilitation found in the lab may be less relevant for actual language learning. More broadly, if CDS is not Zipfian, this could undermine the role of learnability pressures in the emergence of Zipfian distributions. For both reasons, it is important to ask whether CDS is Zipfian or not.

What reason is there to think that CDS will not follow a Zipfian distribution? While CDS is similar in certain aspects to adult-to-adult speech, some of its’ unique and well-documented properties may lead word distributions to be less skewed. In particular, CDS has a smaller lexicon than adult-to-adult speech (Fernald & Simon, 1984; Roy et al., 2015), which may lead to a more uniform distribution of types. CDS also includes many repeated sequences, such as variation sets (where consecutive sentences have overlapping words, e.g., “Where is the bunny? Here is the bunny”; Brodsky et al., 2007; Tal & Arnon, 2018), and recurring frequent frames (Cameron-Faulkner et al., 2003; Mintz, 2003). These differences could lead to word distributions that have fewer types and more similar frequencies across ranks, in contrast with the steep change in frequency that is a hallmark of Zipfian distributions. That is, the lexical and structural differences between CDS and adult-to-adult speech could theoretically lead to a word distribution that differs from the Zipfian one. In such a scenario, word distribution might change over time, becoming “more Zipfian”: Several aspects of CDS, such as mean length of utterance and type-token ratio, change across development and become more “adult-like” with age (Roy et al., 2009). Word distributions may show a similar trend, starting with a less skewed distribution and becoming more Zipfian as the child grows and their vocabulary expands.

What do we currently know about word distributions in CDS? Interestingly, there is little research on this question. Even though Zipfian distributions are assumed to be a basic property of natural language, the existing findings documenting them are based on adult-to-adult speech (most often written language), where both speakers and listeners are proficient language users. To our knowledge, only few prior studies explored the distribution of words in the input directed to young children (Dale & Spivey, 2006; Hendrickson & Perfors, 2019; Lavi-Rotbain & Arnon, 2022; Montag et al., 2018). Montag et al. (2018) focused on whether the amount of the available input or its diversity are more important for learning. One of the measurements the authors looked at was the distribution of words. The authors found that samples of 50,000, 20,000 and 2,000 words in CDS had a skewed frequency distribution that was close to linear on a log-log scale. Hendrickson and Perfors (2019) focused on the facilitative effect of Zipfian distributions for cross-situational word learning in an experimental setting. In the appendix, they provide an analysis of the frequency distribution of nouns in a corpus of English child-directed speech, as a validation of the use of this skewed distribution in the experimental setting. For this dataset, they find that English nouns in CDS follow a near-Zipfian distribution (as reflected in a linear relationship between log frequency and log rank). Dale and Spivey (2006) analysed the correlations of different linguistic measures between child’s and care-giver’s utterances within conversations by using three English corpora from CHILDES (MacWhinney, 2000). One of these measures was frequency distributions of *n*-grams (for *n* = 2, 3 and 4). They showed that on a log-log scale there is a linear relation between the n-grams frequency and their rank (Dale & Spivey, 2006). Lavi-Rotbain and Arnon (2022) looked at the effect of skewed frequency distribution on speech segmentation. First, they analysed corpora of CDS using measures from Shannon’s information theory and found that across languages CDS has similar entropy levels, meaning it is similarly skewed. Then they used these values to create artificial language distributions (Lavi-Rotbain & Arnon, 2022).

Importantly, in these studies, analysing the frequency distribution of words was not the main focus of the study, leaving many open questions. We do not know whether a similar distribution holds for the entire lexicon, across languages, across development and for different parts of speech. In addition, the fit to the Zipfian distribution was illustrated by finding a close fit to a linear distribution on a log-log scale. While informative, this does not necessarily indicate that the original distribution is a Zipfian one, since several different distributions appear linear on a log-log scale (Clauset et al., 2009).

### The Current Study

In the current study, we expand on the existing literature by exploring word distributions in child-directed speech across 15 languages with three goals in mind. The first, and primary one, is to see if words follow a Zipfian distribution in child-directed speech across different languages (Study 1). Such a finding would support the generality of Zipfian distributions in language and their possible role in language learning. We can also use this data to see whether the parameters of the Zipfian distribution - α and β - are similar across languages. A previous study estimated the parameters of the Zipfian distribution based on Holy Bible translations across a large number of languages and found differences across languages (Mehri & Jamaati, 2017). However, they looked at written language, rather than spoken, and tried to fit only the middle region of the distribution, excluding the most and least frequent words (Mehri & Jamaati, 2017). We will ask whether a similar variation will be found in CDS. The second goal is to ask whether there is a developmental change in the distribution of words in child-directed speech - as was found for other structural properties - or whether the distribution is Zipfian from the very start (Study 2). Finally, we want to see if words follow a Zipfian distribution in child-directed speech across different parts of speech (Study 3), as was found for adult-to adult speech (Piantadosi, 2014).

We decided to look at four parts of speech: nouns, verbs, adjectives and prepositions. The skewed distribution of content words like nouns, verbs and adjectives, can be attributed in part to meaning: We talk with infants more about teddy-bears, for example, than stocks. While this does not explain the particular way frequency decreases, it could explain the presence of few highly frequency words and the long tail of the distribution. However, finding such a distribution for prepositions would suggest the impact of factors beyond meaning on frequency for several reasons. First, prepositions are not expected to vary so much in their real-world frequency: there is no reason to think people talk more about objects being “on” other object than “behind” other objects. In addition, different prepositions can be used to describe the same event: We can describe things as being placed ‘under’ or ‘above’, or happening ‘before’ or ‘after’, and so on, meaning that the presence of an event is not fully predictive of the preposition used to describe it. Finally, and most importantly, the meaning of prepositions varies across languages, with languages carving up the meaning space differently (e.g., Bowerman & Choi, 2001; Christiansen & Chater, 2008; Levinson et al., 2003). For instance, the difference between ‘in’ and ‘at’ in English is not reflected in Hebrew where the same preposition is used for both meanings. That is, despite the physical world being the same, the choice of prepositions differs across languages, meaning the world itself cannot be the leading cause for finding a Zipfian distribution in the use of prepositions.

We explore the first question by asking how closely word distributions adhere to Zipf’s law in CDS across 15 languages from seven language families. We address the second question by analysing word distributions across development for the five languages for which we have large enough longitudinal corpora. We address the third question by analysing word distributions across different parts of speech for the five languages for which we have morphologically tagged data. Together, the studies aim to provide a comprehensive assessment of word distributions in CDS. To pre-empt the results, we find that word distributions follow a near-Zipfian distribution across languages, across development and across parts of speech illustrating their prominence in children’s learning environment.

## STUDY 1: DO WORDS IN CDS FOLLOW A ZIPFIAN DISTRIBUTION ACROSS LANGUAGES?

We analysed the distribution of words in CDS for all the languages whose corpora on CHILDES (MacWhinney, 2000) matched our selection criteria (see details below). This resulted in the analysis of 15 languages, from seven language families: **Germanic** (including English - British and North-American, German, Dutch, Swedish, Danish and Norwegian); **Latinate** (including French, Spanish, Portuguese and Catalan); **Uralic** (Estonian); **Slavic** (Polish); **Semitic** (Hebrew), **Japonic** (Japanese), and **Sino-Tibetan** (Mandarin), (see Table 1 for more information about the corpora). We performed two analyses: First, we looked at the entire corpora available for each language and tried to fit it to a Zipfian distribution in order to see if CDS is Zipfian across languages. We then used small samples from languages with large enough corpora to assess the stability of our estimates. This additional analysis allowed us to also ask how similar the parameters of the Zipfian distribution are across languages, or whether they differ between languages, as was previously found for the Holy Bible translations (Mehri & Jamaati, 2017).

**Table 1.** Summary of corpora measures across languages for Study 1.

**Language**	**Child’s Age Range**	**No. Corpora**	**No. Tokens**	**No. Types**	**Word Frequency (per million)**	**α**	**β**	**Pearson’s-*r***
British English	0;2–3;6	12	6311249	27476	1 - 303005 (0.16 - 48010.31)	1.57	19.48	0.97
North-American English	0;5–3;6	34	4876774	24573	1 - 230355 (0.21 - 47235.12)	1.52	18.34	0.965
German	0;5–3;6	7	2168002	37018	1 - 71238 (0.46 - 32858.83)	1.42	12.69	0.998
French	0;11–3;6	8	1540284	19327	1 - 59852 (0.65 - 38857.77)	1.53	17.98	0.99
Dutch	1;5–3;6	5	1036586	18717	1 - 41646 (0.96 - 40176.12)	1.48	12.63	0.998
Japanese	0;6–3;6	6	941006	25648	1 - 45886 (1.06 - 48762.71)	1.30	7.12	0.993
Polish*	0;10–6;11	8	794183.7	44425	1 - 32172.19 (1.26 - 40509.76)	1.16	4.04	0.997
Spanish	0;11–3;6	12	353104	10057	1 - 13437 (2.83 - 38053.94)	1.39	9.96	0.994
Swedish	1;0–3;6	2	341280	9466	1 - 16924 (2.93 - 49589.78)	1.40	7.58	0.998
Portuguese	1;5–3;6	2	309296	7562	1 - 21416 (3.23 - 69241.12)	1.37	5.44	0.981
Hebrew	0;8–3;6	6	300766	13801	1 - 16048 (3.32 - 53357.09)	1.19	3.69	0.996
Norwegian	1;1–2;9	2	183658	8306	1 - 9135 (5.44 - 49739.19)	1.32	6.92	0.996
Estonian	0;9–3;6	5	167666	10057	1 - 10344 (5.96 - 61694.08)	1.21	6.02	0.956
Danish	0;11–3;5	1	155826	4102	1 - 8421 (6.42 - 54041.05)	1.50	7.71	0.997
Mandarin	1;8–2;3	2	150852	7095	1 - 6305 (6.63 - 41795.93)	1.42	11.94	0.989
Catalan	1;1–4;2	4	132410	6416	1 - 8051 (7.55 - 60803.56)	1.30	6.27	0.985
Summary						Mean = 1.38; *SD* = 0.13	Mean = 9.86; *SD* = 5.13	Mean = 0.988; *SD* = 0.013

* For Polish the data is taken from a list including a summary of words and their relative frequencies. Hence the one digit precision.

### Methods

We included all the corpora available in these languages with the following restrictions: the data was collected during parent-child interactions (as opposed to investigator-child or peer interactions); from typically developing children who were 3;6 years old or under during the recording. We only looked at younger ages since we wanted to focus on the input to young learners. We extracted the child’s age from the transcripts directly or from the corpora description available on CHILDES. The age criterion was not applied in Catalan since the data was too limited to be screened out by age, nor in Polish since the data we have is a summary of words and their relative frequencies instead of the original transcripts (see Table 1 for details).

In all analyses, we looked only at utterances produced by adults (both care-givers and experimenters) and removed utterances produced by the child. Finally, we only looked at languages that had at least 100,000 tokens after applying the previous restrictions. This minimal threshold was applied to increase the reliability of the estimations of the Zipfian distribution parameters we are calculating (see Equation 1). Previous work has shown that other distributional measures such as entropy are reliable with a corpus of 50,000 tokens and above (Bentz, Alikaniotis, Cysouw, et al., 2017). We chose a more stringent threshold of 100,000 tokens to ensure reliable results. After applying these criteria, we were left with 15 languages: English (British and North-American), German, Dutch, Swedish, Danish, Norwegian, French, Spanish, Portuguese, Catalan, Polish, Estonian, Hebrew, Japanese and Mandarin.

For each language, we assessed words distributions for the entire corpus (collapsing over individual conversations/dyads, as was done in previous studies of adult speech (Piantadosi, 2014). We did not assess word distributions within single conversations because (a) there is no expectation that each individual conversation will follow a Zipfian distribution (in the same way that word frequencies in a single conversation may not reflect their frequency in a language), and (b) because each conversation does not provide enough types/tokens data to accurately estimate the distribution. We started by extracting single word frequencies from all of the available corpora. A word was defined by its orthographic form. While this definition has its limitations (we discuss them further in the discussion), such as treating “dog” and “dog’s” as two independent words, it is the standard definition of a word in the study of Zipfian distributions and in many cross-linguistic corpus studies (e.g., Bentz, Alikaniotis, Cysouw, et al., 2017; Geertzen et al., 2016; Tal & Arnon, 2018). We cleaned the data by removing comments made by the transcriber (see available Python code at https://osf.io/bp62q/). After creating a list of words and their frequencies, we calculated the rank for each word (the most frequent word was ranked 1, the 2^nd^ frequent was ranked 2 and so on). Words with the same frequency were assigned ranks randomly (e.g., if two words appeared 100 times, one was given rank X and the second rank X + 1)^1^.

#### How to Assess Whether Word Distributions Are Zipfian?

After obtaining a frequency distribution of words, we evaluated “how Zipfian” it is. It is not straightforward to estimate whether a particular word distribution is Zipfian. The simplest approach would be to look at the correlation between frequency and rank on a log-log basis and assess how linear this relation is (under a Zipfian distribution it should be linear). This is the criteria used in some studies (e.g., Hendrickson & Perfors, 2019). However, since other distributions, beside a power law, are linear on a log-log scale, this cannot tell us if the original distribution follows a power law or not (Clauset et al., 2009). Therefore, another approach is needed. One method, proposed by Clauset et al. (2009) involves estimating the minimal rank from which the power-law distribution is supposed to hold; estimating only alpha and then measuring the maximal distance between the observed distribution and the expected power-law distribution using Kolmogorov–Smirnov statistic. This method assumes that word distributions follow a true power-law. We opted not to use it to assess the fit for several reasons: (a) word distributions in language are only nearly-Zipfian, diverging from from the expected Zipfian distribution in systematic ways (Piantadosi, 2014), (b) Clauset’s method has not been applied broadly to linguistic data (Clauset et al only use it to analyze Moby Dick in English) and we wanted our investigation of child-directed speech to be comparable to existing analyses of the fit to the Zipfian distribution in adult speech, and (c) most importantly, we conducted several analyses of our data using this method, which suggest that it generates inconclusive results that do not reflect meaningful variation (see Appendix B). Instead, we opted to use the method used in previous studies of adult speech (Piantadosi, 2014) where the Zipfian nature of the distribution is assessed by (1) estimating the parameters of the distribution (as described below, Equation 1), (2) finding the expected frequency distribution based on the estimated parameter values, and (3) evaluating the goodness of the fit between the observed frequency distribution and the expected one under a Zipfian distribution (Piantadosi, 2014).

Here, we use the same method. We start by estimating the parameters of the Zipfian distribution (see Equation 1). In this equation, *r* is the word’s rank; α is the exponent of the power law while β is a correction added to the original Zipf’s law by Mandelbrot to create a better fit to actual language data (Mandelbrot, 1953). The sign “∝” indicates proportionality: f(r) is proportional to the right-hand side of the equation.

$$f\left(r\right)\propto \frac{1}{{\left(r+\beta \right)}^{\alpha }}$$ (1)

Our application differs from that of Piantadosi (2014) only in that we did not use the split-to-half method where the corpus is divided into half, with one half used to assess frequency and the second used to assess rank. This is claimed to reduce the dependency between the measures of rank and frequency. However, we did not use it here since we did not want to reduce the number of languages we can look at. We need at least 100,000 tokens to reliably estimate the parameters, and did not want to exclude corpora with fewer than 200,000 tokens (we would have to remove five of the languages we currently use). To validate our use of the entire sample, we conducted the split-to-half method for the ten languages for which there was enough data, showing that the parameter estimates and the fit to a Zipfian distribution are almost identical when using this method and using the entire sample (Pearson’s-*r* between the alpha based on the entire sample and the alpha estimated using the split-half method = 0.999; Pearson’s-*r* for beta from the entire sample and the split-half method = 0.999, see Appendix A for details).

In order to estimate the α and β that give the best fit of the data to Equation 1, we used the maximum likelihood estimator (MLE, using the following code in R: https://osf.io/bp62q/), which is a commonly-used algorithm to solve parameter estimation problems (Linders & Louwerse, 2020; Piantadosi, 2014). Finding α and β allows us to (1) estimate the fit of a Zipfian distribution to the data, and (2) see if they are similar across the different languages, despite differences in corpus size, lexicon size, and morphological complexity. For each language, we find the expected Zipfian distribution, based on the α and β values we found. We used the formula for the probability mass function of a Zipfian distribution to calculate the expected frequency (see Equation 2): multiplying each probability by the total number of tokens in the language.

$$p\left(r\right)=\frac{1}{{\left(r+\beta \right)}^{\alpha }}*\sum_{r=1}^{N}\frac{1}{{\left(r+\beta \right)}^{\alpha }}$$ (2)

We correlated the observed frequencies with the expected frequencies using Pearson correlations and obtained the Pearson’s-*r* of the correlation. Note that the correlation we report is the one between observed and expected frequency, and not between frequency and rank: We want to know how much of the observed frequency can be explained by the predicted frequency based on a Zipfian distribution (and not how much the change in rank explains the change in frequency). While α and β were estimated from the data, they are not expected to predict the observed data perfectly. As in any attempt to explain real-world behaviour, there is a certain degree of noise in our estimates. By calculating the fit between the observed (real) distribution and the expected one (generated by plugging the α and β we found into Equation 2), we can see how close they are to each other. In other word, we can see how “Zipfian” the observed distribution is. We present graphs of the observed and expected frequency given rank on a regular and a log-log scale (Figure 1 and 2 correspondingly). The regular scale shows the skewness of the distribution, the sharp decrease in frequency, and the long tail for the least frequent words. The log-log plots show the linear relation between log(rank) and log (frequency).

We wanted to check the reliability of our estimates and also to see if the parameters of the distribution will differ across languages in CDS, as was found for bible translations (Mehri & Jamaati, 2017). To do this, we divided the full corpora into smaller samples of 100,000 tokens. We only examined languages that provided at least three samples of this size, so we would have a reasonable number of data points. This resulted in the use of nine languages from four language families (British English, Dutch, German, Swedish, French, Portuguese, Spanish, Japanese and Hebrew). We did not use North-American English even though it had a large enough corpus, since we did not want too many English samples (British alone had 69 samples). We created the small samples for each language by reading each transcription file from beginning to end until a sample of at least 100,000 tokens was created. This way the samples were conversationally continuous. For each sample we estimated α and β as described above. This yielded several estimations of each parameter for each language, which enabled us to see whether the parameters from the entire corpus were similar to those from the sub-samples, and to compare our estimations within and across languages.

### Results

#### Is CDS Zipfian Across Languages?

The observed and expected distributions for each language are plotted in Figure 2. In these plots, we can see that the two curves are close to one another, indicating that the observed distribution is very close to the expected Zipfian one. This is reflected in the high and significant Pearson’s-*r* for the fit between the two, indicating that α and β we found provide a good fit to the data^2^. That is, the distribution of words in CDS is very close to a Zipfian distribution across languages.

#### Are Word Distributions in CDS Similar Across Languages?

To see how similar word distributions are across languages, we first compared the parameter values we estimated based on the full corpora for each language to the other languages. These parameters, and especially α, directly affect the shape of the distribution. β affects the distribution less and is not expected to be similar across language. However, since α is the exponent, it dictates the slope of the curve: For larger values of α, the resulting distribution will be steeper with a faster decrease in frequency along the rank axis. A similar α across languages, will suggest that word distributions show a similar decrease in frequency, resulting in a steady difference in frequency along the slope.

**Figure 3.**
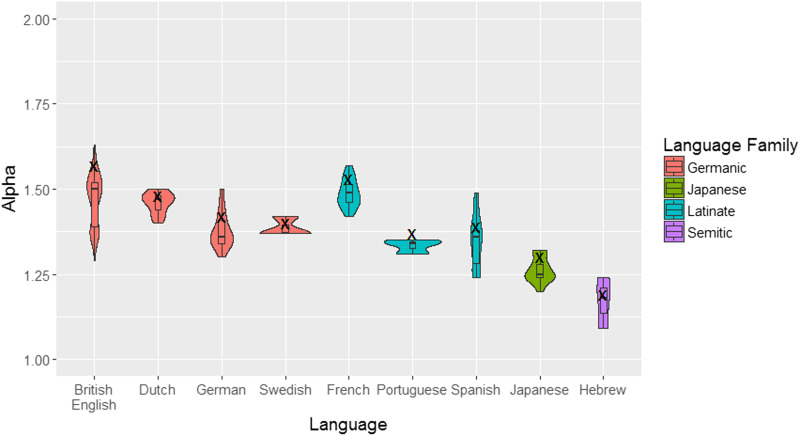
**The estimated α by language.** Color represents language family. Boxes indicate quartiles. “X” represents α value obtained based on the full corpora.

When we look at these values, we see that α has a small SD across languages compared to β (α: mean = 1.38, *SD* = 0.13, range = 1.16–1.57; β: mean = 9.86, *SD* = 5.13), suggesting that the decrease in frequencies by rank is relatively similar across languages. For example, for Catalan we received the same α as for Japanese (α = 1.3) even though our Catalan corpus had only 130,000 tokens and 6,400 types, while the Japanese corpus had over 900,000 tokens and over 25,000 types. To see whether α is similar across languages and language families, we estimated the parameters again using smaller samples of 100,000 tokens each (as described in the Methods section). We generated a total of 149 samples with the following division across languages: 69 for British English; 21 for German; 19 for French; 14 for Japanese; 11 for Dutch; 6 for Spanish; and 3 for Portuguese, Hebrew and Swedish. We estimated the parameters for each sample and plotted the results per language (Figure 3). A one-way ANOVA showed that the effect of language on α is significant (*F*(8, 140) = 28.26, *p* < 0.001), as well as the effect of language family on α (*F*(3, 145) = 36.03, *p* < 0.001). Both effects remained significant after excluding Hebrew that has the lowest α. In addition, α differed across languages from the same language family: the effect of language was significant also when looking only at the four Germanic or at the three Latinate languages (Germanic: *F*(3, 100) = 11.02, *p* < 0.001; Latinate: *F*(2, 25) = 21098, *p* < 0.001). That is, while α spans a relatively small range across languages in CDS, it still shows variation between languages, even ones from the same language family. These results are consistent with the variation found for bible translations across languages (Mehri & Jamaati, 2017), and when comparing word frequency distributions for the Swadesh list across languages (Piantadosi, 2014).

To examine the stability of the parameters we estimated for each language based on the entire corpus, we wanted to compare them to the α estimates based on the sub-samples. Finding that the parameters are similar, would validate the use of the smaller samples for comparing α across languages, and for estimating α from relatively smaller corpora (each of our sub-samples had only 100,000 tokens). To explore this, we compared α calculated for the entire corpora to the range of α values estimated for the sub-samples. For visualisation, we added to Figure 3 the α from the entire corpora (represents by “X”). The correlation between the two is very high (Pearson’s-*r* = 0.98). Interestingly, for all tested languages, the α calculated based on the full corpora is slightly higher than the ones averaged across samples (e.g., for Hebrew α for full corpora is 1.19 while the average of the three smaller samples is 1.17). However, the values for the entire corpora fall within the range obtained from the different samples for all languages except Portuguese (see details and more discussion of the relation between α and corpus size in Study 2, Figure 5)^3^.

#### Is the Word Distribution in CDS Similar to That of Adult-to-Adult Speech?

**Figure 4.**
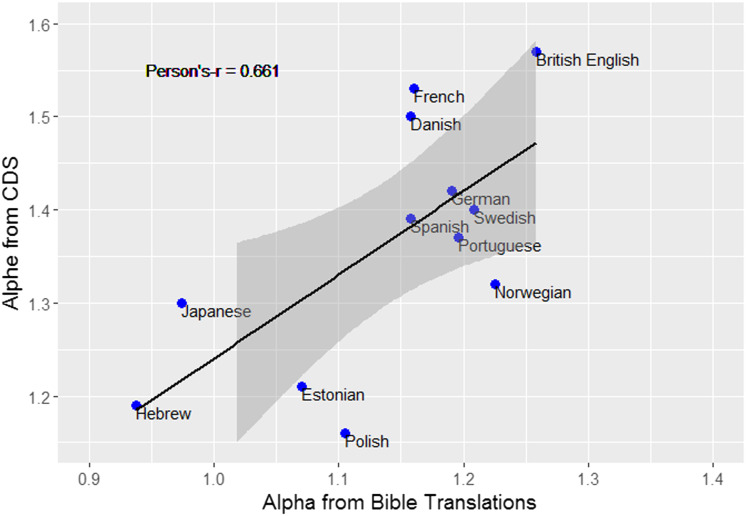
The correlation between α calculated based on CDS (*y*-axis) and α calculated based on bible translations for 12 languages (*x*-axis, taken from Mehri & Jamaati, 2017, Table 1, p. 2473).

Another interesting comparison is between the parameters of the distribution obtained for CDS and for adult-to-adult speech. The α values found here are generally similar to what was previously found for adult-to adult speech, though there is large variation between different adult corpora. For example, based on the American National Corpus (Reppen & Ide, 2004), Piantadosi reports that α = 1.13 (Piantadosi, 2014). However, other adult-to-adult spoken and written English corpora yielded higher values for English (range = 1.728–1.940; Arnold, 2015, page 14; α = 1.95 for words appearing in Moby Dick; Clauset et al., 2009). Putting aside the variation between different corpora of the same language, the previous section showed differences in α across languages (e.g., English has the highest α while Hebrew has the lowest). We can ask if α varies in similar ways across languages in both adult and child-directed speech. If so, it can suggest that the variation is driven by linguistic properties of the languages shared by all speech registers (such as morphological complexity or lexicon size). To examine this more closely, we compared the α we received based on the entire corpus to the ones generated from bible translations (Mehri & Jamaati, 2017, Table 1, p. 2473). There were 12 overlapping languages between the studies, with two estimations for each (Figure 4). Note that there are differences in how α was assessed in each of the studies: In our study we tried to fit the entire distribution to Equation 1 (including both α and β as parameters), while Mehri & Jamaati tried to fit only the middle portion of the distribution to the simplified version of Zipf’s law (including only α). Interestingly, despite these differences, the correlation between the two α values is positive and significant. Languages that have a higher α based on bible translations also have a higher α in CDS (*n* = 12, Pearson’s-*r* = 0.67, *p* < 0.05), suggesting the variation is impacted (at least in part) by linguistic/structural properties of the language itself. In the next study, we ask whether the good fit to a Zipfian distribution is found across development.

## STUDY 2: DOES CDS FOLLOW A ZIPFIAN DISTRIBUTION ACROSS DEVELOPMENT?

In Study 1, we found that CDS follows a Zipfian distribution when we collapse across ages. However, it is possible that the skew is driven by speech directed to older children. To test this, we now analyze the distribution of words in CDS separately for different ages. This enabled us to see if the shape of the distribution is already found in infancy or whether it changes over time, like other linguistic measures that become more “adult-like” as the child grows (Roy et al., 2015). We only looked at languages where there was sufficient data to divide into age bins, and that had an age range that started in early infancy. We also wanted to look at languages from diverse language families. Based on these two parameters, we focused on five languages: English (British), German, French, Japanese and Hebrew.

### Methods

For each language, we divided the available transcripts into fixed age bins of six months, ranging from birth up to five years old (i.e., the 1^st^ bin included all available data between 0;0–0;5 months, the 2^nd^ bin included 0;6–0;11 months and so on). Because the available data in each language began and ended at different ages (i.e., the youngest available age in French was eleven months, while in English it was 2-months), we could not look at the entire range for all languages (see Table 2 for details). For each age bin, we plotted the observed distribution of words (Figure 6).

**Table 2.** Summary of corpora measures across development for Study 2 for British English.

**Child’s Age Range**	**No. Tokens**	**No. Types**	**Word Frequency (per million)**	**α**	**β**	**Pearson’s-*r***
0;2–0;5	5672	694	1 - 336 (176.3 - 59238.36)	–	–	–
0;6–0;11	15011	1147	1 - 735 (66.62 - 48964.09)	1.42	16.43	0.969
1;0–1;5	24047	2954	1 - 1916 (41.59 - 79677.3)	1.14	3.18	0.96
1;6–1;11	222896	5627	1 - 9309 (4.49 - 41763.87)	1.54	18.33	0.984
2;0–2;5	2432536	15012	1 - 116879 (0.41 - 48048.21)	1.55	18.38	0.971
2;6–2;11	2276518	16728	1 - 110405 (0.44 - 48497.31)	1.54	18.15	0.971
3;0–3;5	1272241	12695	1 - 62026 (0.79 - 48753.34)	1.52	17.42	0.972
3;6–3;11	321097	8176	1 - 12771 (3.11 - 39773.03)	1.46	16.4	0.979
4;0–4;5	262627	7694	1 - 10464 (3.81 - 39843.58)	1.44	14.9	0.982
4;6–4;11	186765	6375	1 - 7288 (5.35 - 39022.3)	1.41	13.56	0.988
Summary				Mean = 1.49; *SD* = 0.06	Mean = 16.73; *SD* = 1.88	Mean = 0.978; *SD* = 0.006

To further analyze the data, we needed to determine the minimal corpus size (in tokens) for which the parameter estimates would be stable. This step was required since the amount of data we had was not uniformly distributed across age: We had more data (in terms of both number of tokens and types) for ages 24–36-months, and less for the other ages (see Figure S1). This inherent bias in our data could influence our variables estimates. We used the largest age bin (2;0–2;6 years old, British English, containing 2.4 million tokens) to find the minimal bound for reliable parameter estimation. We sampled data from this age bin to create samples of varying size: We started from 5,000 words, and increased the sample by 5,000 words until we reached 100,000 words. We created the samples by randomly selecting transcripts, and then reading each transcription file from beginning to end until we reached the required number of tokens. This created samples that were conversationally continuous. We created ten different samples for each sample size (e.g., ten 5,000-word samples, ten 10,000-word samples, etc.)

**Figure 5.**
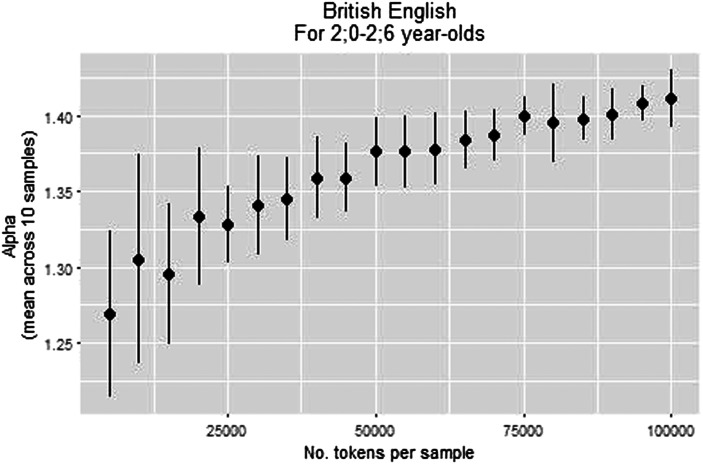
**α by number of tokens per sample.** Each point represents ten samples. Bars indicate the *SD* of the samples.

Figure 5 shows the change in α as a result of changing the sample size: α increased with sample size, and began to stabilize at 50,000 tokens (as reflected in the small SD of 0.02 and the smaller changes in its value). This finding is in the same direction as in Study 1: α calculated on the full corpus was slightly higher than α calculated on samples of 100,000 tokens (Figure 3). While in Study 1 we chose a minimal size of 100,000 tokens per sample, here we could not choose such a stringent minimal bound since it would have left us with too little samples. Therefore, we chose 50,000 tokens as our minimal bound. This left us with thirty usable age bins.

For age bins with at least 50,000 tokens, we conducted the same analyses as in Study 1: (1) evaluating α and β; (2) calculating Pearson’s-*r* between the observed and the expected distributions; (3) plotting the two curves together. We used mixed effect regression models to see if α or Pearson’s-*r* are affected by age. Specifically, we ask two questions. The first, does the distribution become more Zipfian during development, or is it near-Zipfian from the start? To test this, we looked at whether Pearson’s-*r* changes across the different age bins, and whether it is high even for the youngest age bins. The second question was whether the slope of the distribution changes across development. Such a change would indicate that the distribution becomes more or less skewed as the child grows. To test this, we looked at whether α changes across the different age bins.

### Results

**Figure 6.**
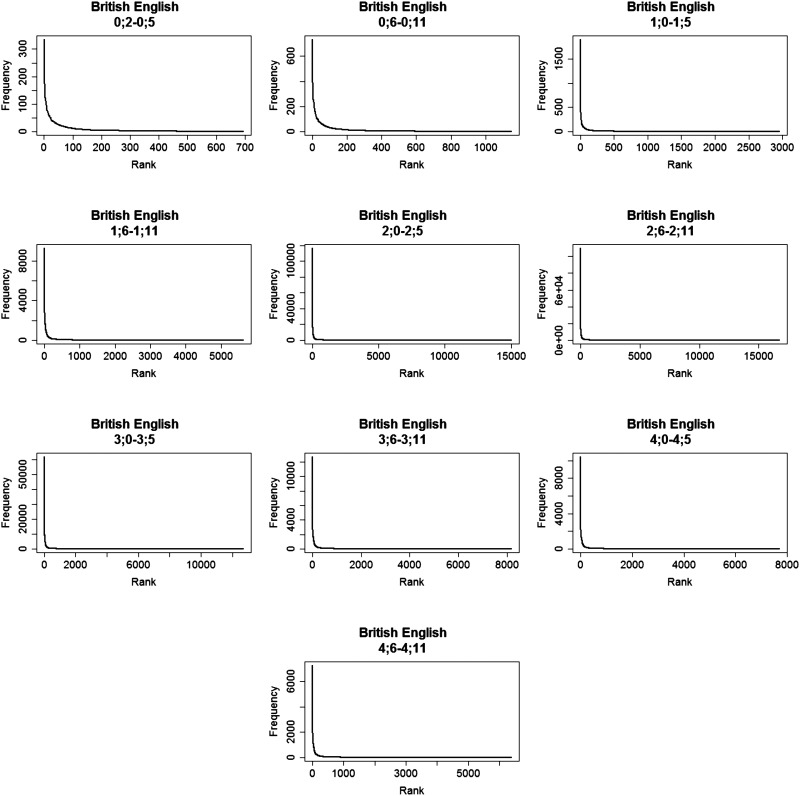
**Word distributions in British English across development, in bins of six months.** Note that the age bin of 0;2–0;5 did not pass the minimal size requirement of 50,000 tokens, but still shows a highly skewed distribution.

**Figure 7.**
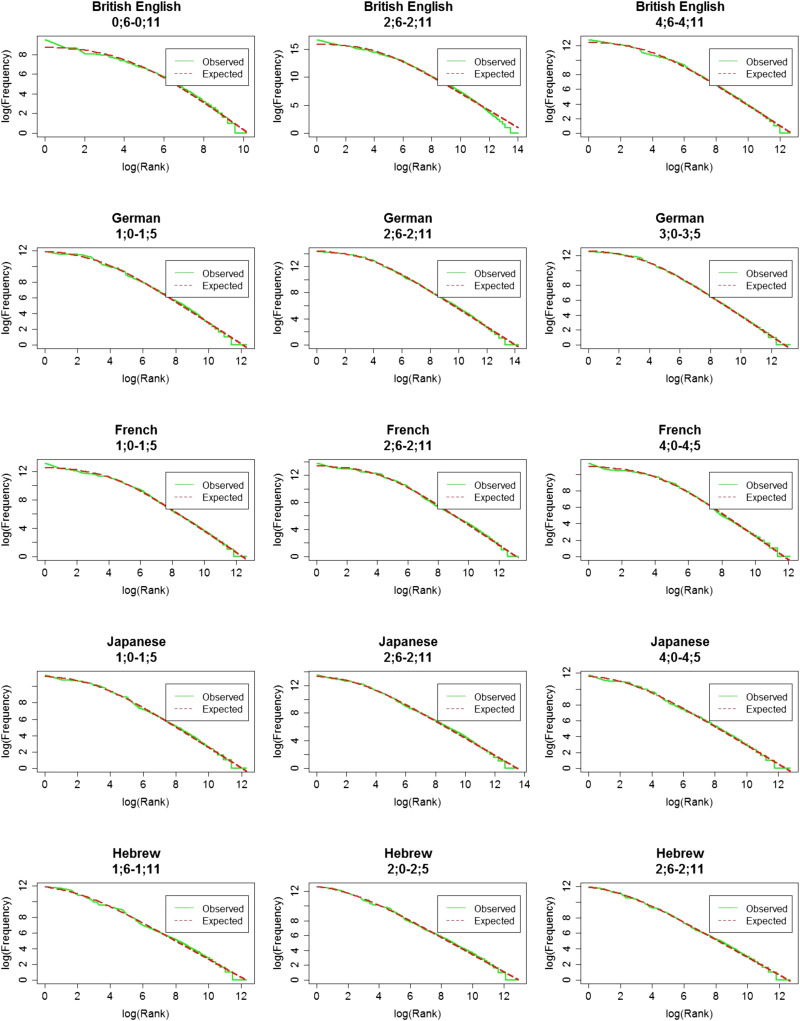
**Observed (in green) and expected (in red) word distribution on a log-log scale.** Three age bins per language are presented. All age bins here passed the minimal size restriction of 50,000 tokens.

Due to lack of space, we only show the results for British English (Table 2), where we had the largest age range (the full results are provided in Table S1 and Figure S1–S5 in the Supplementary section). Figure 6 shows word distributions for British English across development. Both Table 2 and Figure 6 include all age bins for British English, including the one containing less data than our minimal bound. All age bins (even the age bin of two-to-five months that included only 5,000 tokens), followed a skewed frequency distribution. This held for all age bins in the other languages as well, meaning that even small samples of CDS are skewed. Thirty age bins passed the minimal size restriction (twelve were removed since they were too small) and were further analyzed. Figure 7 shows the observed and expected word distributions of the age bins that passed the minimal size restriction (three age bins per language, other bins appear in Figure S6–S10). Importantly, there was a good fit between the observed frequency distribution and the expected Zipfian distribution for all age bins, reflected in a very high Pearson’s-*r* (see Table 2 and Table S1 for details).

**Figure 8.**
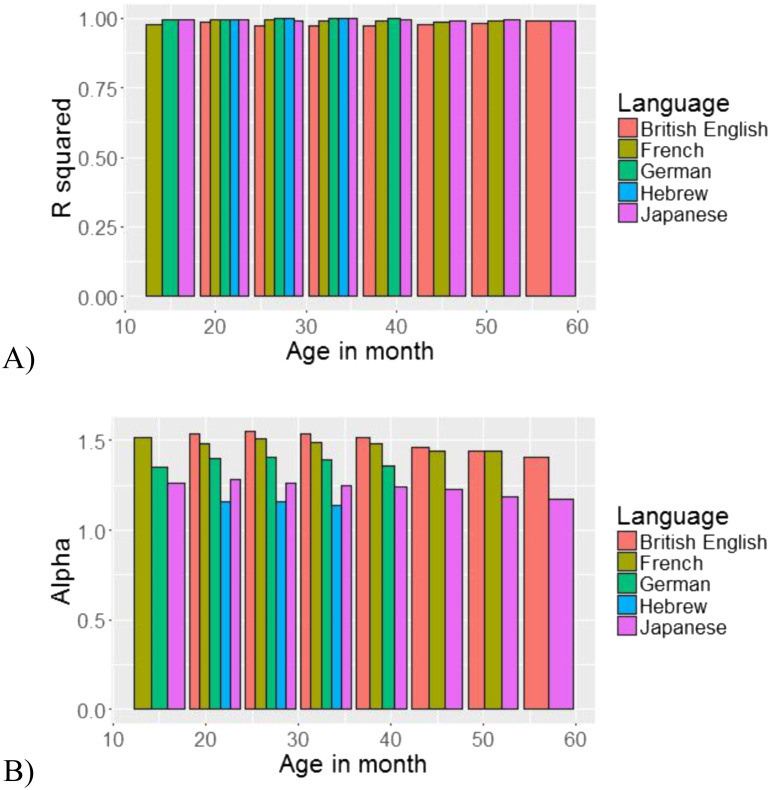
(A) Pearson’s-*r* and (B) α as a function of age.

We wanted to further ask whether the distribution becomes “more Zipfian” as the child grows (has higher Pearson’s-*r*), and whether the slope of the distribution changes with age. To answer these questions, we ran two mixed-effect regression models. Following Barr et al. (2013), the models had the maximal random effect structure justified by the data that would converge. In the first model, our dependent variable was Pearson’s-*r*; age (in months, centred) was our fixed effect, and we had a random intercept for language as well as by-age random slope for the effect of language (Table 3). Age was defined as the minimal age per bin (e.g., for an age bin of 1;0–1;5 years, age for the regression was 12 months). While CDS follows a Zipfian distribution from the very start, it does not become “more Zipfian” with age: Pearson’s-*r* did not change with age (β = 0.00004, *SE* = 0.00008, *p* > 0.6). This trend can be seen in Figure 8A: Pearson’s-*r* is significantly high throughout development, without any visible change. The second model had the same fixed and random effects as the previous one, but this time α was our dependent variable (Table 4). Here we saw a minor effect of age on α: The slope of the distribution became somewhat more moderate across development, as α decreased with age (β = −0.0021, *SE* = 0.0007, *p* = 0.062, Figure 8B). While this effect did not reach significance, it may indicate that speech directed to younger infants is more skewed than speech directed to older children.

**Table 3.** Mixed-effect regression model looking at Pearson’s-*r* as a function of age. Variables in bold were significant. Significance obtained using the lmerTest.

	**Estimate**	**Std. Error**	***df***	***t* value**	***p*-value**
**(Intercept)**	9.903e−01	3.631e−03	3.982e+00	272.740	<.001***
Age (in months)	4.395e−05	8.249e−05	6.098e+00	0.533	>0.1

**Table 4.** Mixed-effect regression model looking at α as a function of age. Variables in **bold** were significant. Significance obtained using the lmerTest.

	**Estimate**	**Std. Error**	***df***	***t* value**	***p*-value**
**(Intercept)**	1.3511334	0.0701455	3.9882635	19.262	<.001***
**Age (in months)**	−0.0020710	0.0007186	3.0599781	−2.882	**=0.062**

Study 2 showed that CDS is skewed from the very start, and throughout development. This skewed nature is seen in all of our samples, including the ones that did not reach the minimal size requirement. In addition, while CDS does not become more Zipfian, its exponent becomes mildly smaller with age, suggesting the contrast between high and low frequency decreases somewhat with age (see Discussion). In the next study, we will ask if different parts of speech also show such a skewed frequency distribution.

## STUDY 3: DOES CDS FOLLOW A ZIPFIAN DISTRIBUTION ACROSS PARTS OF SPEECH?

In the third study, we ask whether different parts of speech in child-directed speech will also show a Zipfian distribution, as was previously found for adult directed speech (Piantadosi, 2014; Ramscar, 2020). We looked at the following five languages - British English, German, French, Spanish and Hebrew – because they all had large morphologically tagged corpora, and represent different language families. We looked at word distributions in these languages across four parts of speech: nouns, verbs, adjectives and prepositions. While the distribution of the first three parts of speech can be attributed, at least in part, to meaning (we talk about certain entities more than on others, or do certain actions more than others), it is hard to attribute a similar real-world meaning to prepositions, because they do not overlap in meaning across languages (e.g., Bowerman & Choi, 2001; Levinson et al., 2003), and because different prepositions can be used to describe the same event. Finding that prepositions also follow a Zipfian distribution will emphasize the role of pressures beside meaning – such as communicative efficiency or learnability – in the propensity of such skewed distributions in language (see Discussion for more on this).

### Methods

We used all the available corpora in British English, German, French, Spanish and Hebrew. We used the morphological tagging provided in the corpora to automatically extract the relevant words (nouns, verbs, adjectives, and prepositions). However, we wanted to make sure the tagging in the corpora was accurate. To examine this, all the data was manually examined by research assistants, who were proficient speakers of the languages in question. The research assistants were asked to go over the extracted lists for each POS, and remove the words that did not belong to that part of speech. The words were not added to their correct part of speech list, since the part of speech for many words cannot be fully determined out of the context (i.e., “run” can be either a verb or a noun) and we did not want to introduce additional noise. After the manual cleaning, we calculated the error rate per language and per POS by dividing the number of types that were mistakenly tagged in each category by the total number of types in each category (e.g., 37 types erroneously classified as Spanish adjectives out of a total of 754 Spanish adjectives). Error rates were very high for prepositions across all languages, but much lower for nouns, verbs and adjectives (see Table 5 for full details). We do not know why the error rates are so high, this may reflect our automatic extraction of the tagged data (rather than errors in the tagged data itself).

**Table 5.** Summary of corpora measures across parts of speech for Study 3.

**Language**	**Part of Speech**	**No. Tokens**	**No. Types**	**Word Frequency**	**Error Rate**	**α**	**β**	**Pearson’s-*r***
**British English**	nouns	1,088,319	17,555	(1 – 17,274)	0.084	1.53	75.52	0.926
	verbs	796,976	3,978	(1 – 46,170)	0.037	1.74	9.93	0.992
	adjectives	226,907	1,987	(1 – 19,925)	0.083	1.6	7.13	0.962
	prepositions	341,910	74	(2 – 58,821)	0.75	8.59	33.97	0.985
**German**	nouns	116,944	10,951	(1 – 13,086)	0.098	0.9	−0.41	0.934
	verbs	56,399	2,660	(1 – 2,986)	0.136	1.54	8.09	0.992
	adjectives	28,388	1,259	(1 – 2,628)	0.36	1.58	8.81	0.962
	prepositions	31,724	38	(1 – 5,817)	0.957	49.77	265.13	0.986
**French**	nouns	169,214	7,305	(1 – 3,347)	0.102	1.35	37.54	0.947
	verbs	189,203	3,844	(1 – 18,234)	0.034	1.46	4.33	0.995
	adjectives	28,517	1,349	(1 – 4,240)	0.017	1.42	3.62	0.927
	prepositions	69,489	46	(1 – 15,717)	0.92	115.07	476.04	–
**Spanish**	nouns	39,155	3,446	(1 – 1,099)	0.075	1.15	11.85	0.974
	verbs	42,059	2,556	(1 – 1,514)	0.006	1.32	8.56	0.991
	adjectives	6,320	716	(1 – 426)	0.0491	1.39	12.06	0.946
	prepositions	12,672	17	(1 – 4552)	0.77	129.8	235.1	–
**Hebrew**	nouns	43,294	2,543	(1 – 2,309)	0.25	1.17	9.47	0.921
	verbs	27,161	2,705	(1 – 1,038)	0.067	1.19	11.89	0.966
	adjectives	6,915	671	(1 – 281)	0.203	1.54	16.32	0.995
	prepositions	23,537	172	(1 – 3,519)	0.687	4.2	18.74	0.986
Summary			nouns	Mean (*SD*)		1.22 (0.24)	26.8 (30.6)	0.94 (0.02)
			verbs	Mean (*SD*)		1.45 (0.21)	8.56 (2.79)	0.99 (0.01)
			adjectives	Mean (*SD*)		1.5 (0.1)	9.59 (4.84)	0.96 (0.025)
			prepositions	Mean (*SD*)		61.5 (58.6) 20.9 (25.1)*	205.8 (188.4) 105.9 (138.1)*	0.99 (0.002)*

* Measured only on English, German and Hebrew.

We used the manually cleaned dataset to calculate the frequency distribution for each part of speech in each language and then did the same analyses as in the previous studies: (1) evaluating α and β; (2) calculating Pearson’s-*r* between the observed and the expected frequency distributions; (3) plotting the two curves together.

### Results

**Figure 9.**
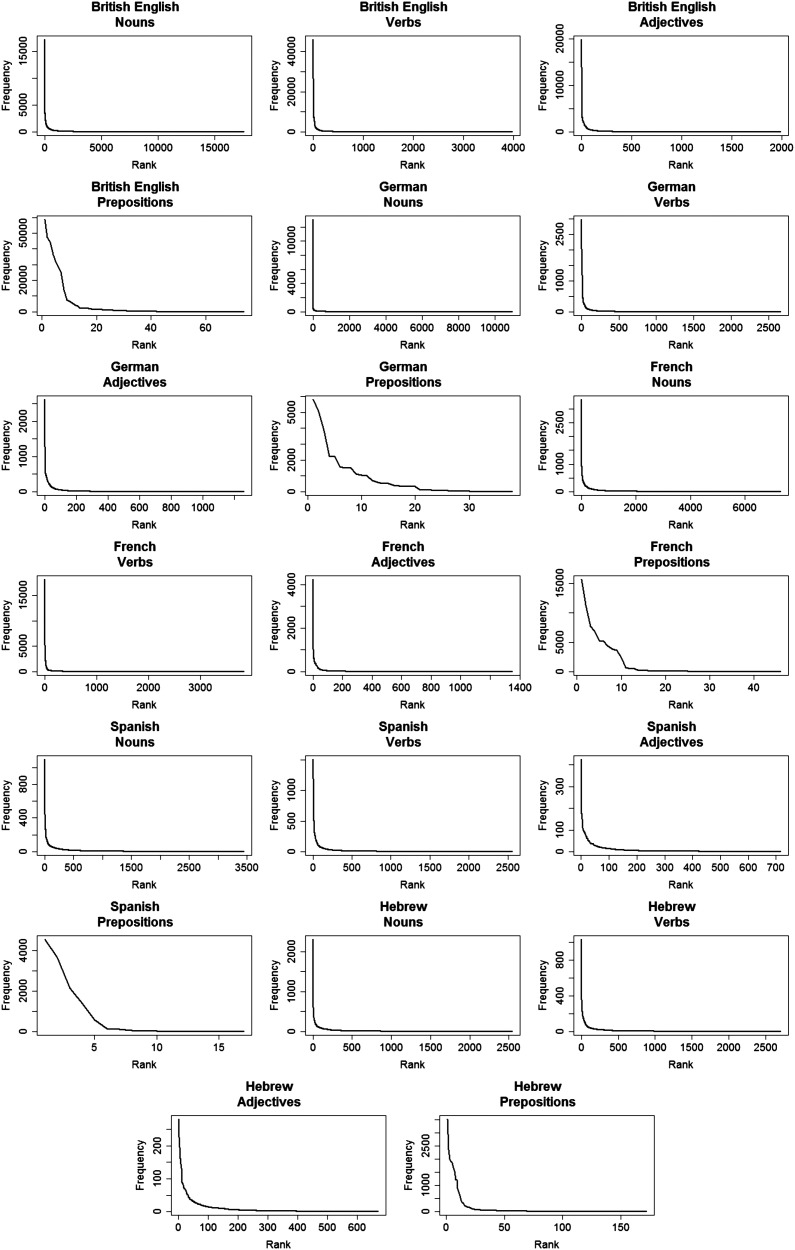
**The observed distributions for four parts of speech across five languages.** Note that Spanish prepositions had to little types to be analyzed further, but still shows a highly skewed distribution.

All four POS in the five languages had a skewed frequency distribution (Figure 9). To ask if this distribution is Zipfian, we estimated the parameters and compared the expected frequency with the observed one. For Spanish and French prepositions, the estimated α was very high (115 and 129.8 respectively) compared to all other estimated α (Table 5). As mentioned, α is the exponent of the distribution, with higher α for more skewed distributions. In this case, the high value of α leads to a binary expected distribution: The most frequent word was assigned a probability of one while the rest of the words were assigned a probability of zero. Therefore, we could not correlate the observed and the expected distributions for Spanish and French prepositions (so they are absent from Table 5).

Beside these two datasets, all tested parts of speech, in all languages, showed a very good fit to the Zipfian distribution, as indicated by the high Pearson’s-*r* scores (Figures S11 in the Supplementary shows results on a log-log scale). Importantly, prepositions in English, German and Hebrew also showed a very good fit to the Zipfian distribution (mean = 0.99). Across parts of speech, α is relatively similar across languages (nouns: mean = 1.22, *SD* = 0.24; verbs: mean = 1.45, *SD* = 0.21; adjectives: mean = 1.5, *SD* = 0.09, Table 5). However, prepositions seem to have a higher α and larger SD compared to nouns, verbs and adjectives (mean = 61.4, *SD* = 58.6, for only English, German and Hebrew: mean = 20.9, *SD* = 25.1). This finding may be related to several unique properties of prepositions: prepositions have the smallest number of types. At the same time, high frequency prepositions are of the most frequent words in the language (looking only at the four categories used here, the most frequent word in each language was a preposition), while the minimum frequency for this group is the same as for the others (1–2 appearances). This means that frequency must decrease rapidly for prepositions, resulting in a higher α. However, it is important to note that the small number of preposition types might impair the reliability of the calculation of α: Our results clearly show that the distribution of prepositions is indeed skewed (see Figure 9), but whether they follow a Zipfian distribution and what are its parameters need to be further examined.

In sum, the examination of the distribution of word frequencies across four POS categories and in five languages shows a strong recurrence of near-Zipfian distributions, and highlights the prevalence of this kind of skew in children’s linguistic environment.

## DISCUSSION

We set out to ask whether words in child-directed speech (CDS) follow a Zipfian distribution (Zipf, 1949). This distribution, where word frequencies follow a power law distribution, is one of the striking commonalities between languages, and has been found across languages (Piantadosi, 2014). However, almost all previous documentation of this phenomenon has focused on adult-to-adult speech, most often written language (Lestrade, 2017; Mehri & Jamaati, 2017; Moreno-Sánchez et al., 2016; Petersen et al., 2012; Piantadosi, 2014; Ramscar, 2020). If this distribution is a hallmark of language, and in particular, if it is driven, at least partially, by learnability pressures (Bentz, Alikaniotis, Cysouw, et al., 2017; Lavi-Rotbain & Arnon, 2022), it should also be found in the input children hear. At the same time, CDS has several unique lexical and structural characteristics, which could lead to a less skewed distribution. Importantly, no study to date has systematically examined word distributions in CDS across languages and parts of speech (but see: Dale & Spivey, 2006; Hendrickson & Perfors, 2019; Lavi-Rotbain & Arnon, 2022; Montag et al., 2018). Here, we expand on current findings to ask whether words follow a Zipfian distribution in CDS across languages, across development, and for different parts of speech. We assessed how Zipfian a distribution is by evaluating the parameters of the power law equation using the maximum-likelihood estimator, and computing the Pearson correlation between the observed and the expected frequency distribution. We find that words follow a Zipfian distribution in CDS across 15 languages from seven language families (Study 1); that they are Zipfian across development, even in speech directed to infants under the age of one (Study 2); and across different parts of speech (Study 3).

What is the importance of these findings? First, these results support the generality of Zipfian distributions in language, showing it is found in another speech register besides adult-to-adult speech. Second, finding that different parts of speech, and specifically prepositions^4^, also follow a Zipfian distribution indicates that Zipfian distributions are not driven only by semantics. Finally, these results highlight the skewed nature of infants’ early learning environments. Our findings join others in showing the skewness of infant’s environment (Clerkin et al., 2017; Lavi-Rotbain & Arnon, 2021, 2022). This skewness might have implications for learning, as suggested by recent studies showing improved learning in such distributions (e.g., Hendrickson & Perfors, 2019; Kurumada et al., 2013; Lavi-Rotbain & Arnon, 2020, 2021, 2022). This learnability advantage is also supported by findings showing that speakers turn uniform distributions into skewed ones in the process of cultural transmission, a pattern consistent with the presence of a cognitive bias for them (Shufaniya & Arnon, 2022). It has been shown that word segmentation is facilitated in language-like distributions. Since segmentation is one of the first challenges infants face when breaking into the speech stream, finding that these distributions are present when infants are segmenting speech strengthens their potentially facilitative role in the process.

The results also show that α – which determines the slope of the distribution - spans a relatively narrow range (1.16–1.57) across languages, highlighting the similarity between them. This relatively narrow range of values raises the possibility that these values of α are more optimal than others for learning, and are therefore preserved across languages. The slope of the distribution dictates how fast frequency will decrease with rank: Steeper slopes will lead to a faster decrease resulting in a larger contrast between high and low frequencies. This might have implications for learning: It has been suggested that the slope found in natural languages is uniquely beneficial for language learning tasks, compared to more moderate slopes (Lavi-Rotbain & Arnon, 2022). At the same time, there is still consistent variation in α across and within language families. Previous studies examining bible translations also found variation across languages (Mehri & Jamaati, 2017; Piantadosi, 2014). Interestingly, it seems that α varies in a similar way across languages in CDS and in the bible translations (Figure 4), suggesting that some of the variation may reflect structural/linguistic differences between the languages. Further research is needed to know which linguistic properties affect α, and in what way.

Methodologically, our findings highlight the importance of using learning environments similar to those of the real world. Despite the prevalence of Zipfian distributions in the wild, most lab-based explorations of word segmentation and learning use experimental paradigms where participants are exposed to uniform distributions, where each novel item appears the same number of times. For example, in the auditory statistical learning task used to assess word segmentation in the lab (Saffran et al., 1996), learners are exposed to novel words in a uniform distribution. While the use of uniform distributions allows researcher to control for frequency effects, they differ drastically from natural input and may lead to difficulties in learning. Interestingly, the presence and utility of skewed distributions is not limited to language: the objects infants see follow a Zipfian distribution (Clerkin et al., 2017), as do object combinations (Lavi-Rotbain & Arnon, 2021). Accordingly, children and adults are better at learning relations between objects (better visual statistical learning) when they are presented in a Zipfian distribution (Lavi-Rotbain & Arnon, 2021). These findings point to the importance of using learning environments that provide a more ecological reflection of children’s learning environment, and the need to further explore the impact of skewed distributions on learning.

One of the limitations of the current study is how we defined what a word is. We defined a word by its orthographic form as is commonly done in investigations of word distributions (Cohen Priva & Gleason, 2016; Petersen et al., 2012; Piantadosi et al., 2011). This means that we treated words such as ‘walk’ and ‘walks’ as two unrelated items. In morphologically rich languages, such as German and Hebrew, this will result in a larger lexicon compared to less morphologically rich languages. For example, while English only has around five verb forms for each verb, Hebrew can have up to twenty separate items for a given verb. That is, defining words orthographically means increasing the number of types more for morphologically rich languages compared to less morphologically rich ones. Defining words in this way also doesn’t account for the semantic relatedness between word forms. The ecological validity of using orthographic form to define a word is supported by evidence that speakers do represent inflected forms, and not only stems and morphemes, and that they are sensitive to whole-form frequency (Baayen et al., 1997). However, further work is needed to see whether the distribution shape or slope change across languages when a different word definition is used. Of particular interest is looking at lemmas to see whether this changes the distribution more for languages with rich morphology.

The results presented here are limited to speech produced by adults: We did not look at the productions of the children themselves for both methodological and theoretical reasons. From a methodological perspective, children at the ages we examined simply do not provide enough linguistic output to reliably estimate the parameters of the distribution. This is especially true for the younger ages where infants often produce only single words. Moreover, caregivers who speak the same language share much of their lexicon, making it is reasonable to combine different conversations into one larger database. However, this may not hold for the younger ages: children who have just begun to speak produce only few words, and these do not always overlap between different infants, making it problematic to collapse frequency counts over different infants. There are also theoretical reasons not to collapse the speech of children at different ages: The kind of language produced by a two-year old (mostly content words: Caselli et al., 1995; Goodman et al., 2008) is very different from that produced by a five-year-old making it hard to understand the patterns of an aggregated distribution. To understand whether the language produced by individual learners is Zipfian and when it becomes so, two interesting and open questions, would require conducting longitudinal analyses of dense individual corpora.

In sum, we showed in this paper that word distributions in CDS are Zipfian across languages, across parts of speech, and from early on. These findings support the generality of Zipf’s law in language and highlight the skewed nature of infant’s early linguistic environment. While many questions remain open, these results strengthen the suggestion that such skewed distribution may be relevant for learning.

## ACKNOWLEDGMENTS

We thank Zohar Aizenbud, Hila Merha and, Maya Ravid for help with extracting and cleaning the data. We thank Shira Tal for her helpful comments. We thank all researchers who made their corpora available through CHILDES. The research was funded by the Israeli Science Foundation grant number 584/16 and grant number 445/20 awarded to the second author.

## Notes

^1^ *We* did not use the split-to-half method proposed by Piantadosi (2014) where the corpus is divided into half by a binomial distribution with one half used to assess frequency and the second used to assess rank (which reduces the dependency between the measures of rank and frequency) since we did not want to reduce the number of languages we can look at (see details on page 15 and Appendix A).^2^ The reported Pearson’s-*r* values were obtained by correlating the observed and expected distribution. In addition, a similar calculation was performed on a log-log scale of these variables, with similar results.^3^ The difference in α may be related to corpus size, for example, larger samples tend to have larger vocabularies, but may also have higher frequencies for the frequent words making the slope slightly steeper. We discuss the relation between corpus size and α further in Study 2 (Figure 5).^4^ While prepositions clearly follow a skewed distribution, the small number of types in the corpora we looked at makes our parameter estimations less reliable than the ones for nouns, verbs and adjectives. More data is needed to establish the fit to a Zipfian distribution.

## APPENDIX A: COMPARING PARAMETERS ESTIMATED USING THE ENTIRE CORPUS AND THE HALF-SPLIT METHOD FOR TEN LANGUAGES

To ensure that our use of the entire corpus to estimate the parameters did not impact the results, we applied the half-split method (Piantadosi, 2014) to the ten languages where we had enough data. We first used a binomial split to cut the data into half: the first half was used to estimate word frequency and the second half was used to estimate its rank, to reduce the dependency between the two measures. The binomial split works as follows: For each word, we sample from a binomial the same number of times as its frequency (with a probability for success – *p* – equals to 0.5) and receive a number (close to half of the overall frequency but not equal to it) that will now be used as the word’s frequency. The difference between the original frequency and the new one is used to assign each word its rank. For example, the word “up” in British English appeared 23,251 times. If we would assign it a rank based on this frequency, it would receive *r* = 55. However, the frequency after the binomial split was 11,731. The frequency for the rank estimation is the difference between the two (23251 − 11731= 11520), which gave it rank 57.

The ten languages we analysed are the ones with at least 200,000 tokens so that each half of the sample has approximately more than 100,000 tokens (depends on the way the data was split). These were: North-American and British English, German, French, Dutch, Japanese, Spanish, Swedish, Portuguese and Hebrew. Table A1 shows the estimated parameters for each language using the full corpus and the split-half method. Importantly, the parameters estimated by the two methods – with and without binomial split – are highly correlated (Pearson’s-*r* for α = 0.999; Pearson’s-*r* for β = 0.999) with α values almost identical between the two methods. β values also show high similarity. This analysis validates our use of the entire corpus to estimate the parameters.

**Table A1.** Summary of corpora measures across languages with and without binomial split.

**Language**	**With binomial split**			**Without binomial split (taken from Study 1)**		
	**α**	**β**	**Pearson’s-*r***	**α**	**β**	**Pearson’s-*r***
British English	1.56	19.21	0.97	1.57	19.48	0.97
North-American English	1.52	18.04	0.965	1.52	18.34	0.965
German	1.41	11.61	0.997	1.42	12.69	0.998
French	1.52	17.47	0.99	1.53	17.98	0.99
Dutch	1.47	11.98	0.998	1.48	12.63	0.998
Japanese	1.29	6.73	0.993	1.30	7.12	0.993
Spanish	1.37	9.34	0.994	1.39	9.96	0.994
Swedish	1.38	7.17	0.997	1.40	7.58	0.998
Portuguese	1.35	5.01	0.983	1.37	5.44	0.981
Hebrew	1.17	3.27	0.996	1.19	3.69	0.996

## APPENDIX B: EXAMINING THE STABILITY OF THE CLAUSET METHOD

We used the ‘poweRlaw’ library (Gillespie, 2015) in R (R Core Team, 2021) to assess the fit of our child-directed corpora and of several adult corpora (ADS) to a true power law. We found that the *p*-value determining if the data was taken from a power-law distribution was not consistent across analyses in ways that do not seem to reflect meaningful variation in the data. In a nutshell, using 100 or 1000 bootstrap iterations, about half of the languages in our sample had a *p* < .05, meaning they deviate from a power-law distribution but whether they deviated did not correspond to our other estimates of the fit to a Zipfian distribution and was not consistent even within the same language (e.g., British English deviated but North-American English did not).

To see if the presence of deviating languages reflects a true difference in word distributions between CDS and ADS, and to further explore the seeming inconsistencies the method generates, we also analyzed two American-English adult speech corpora using the Clauset method (The Santa Barbra corpus; Du Bois et al., 2000) and CABank English CallFriend Northern US Corpus from CHILDES (MacWhinney, 2000), see details below). Applying this method to two comparable adult speech corpora of the same language also led to inconsistent results with one corpus drawn from a power-law (CallFriend from CHILDES, *p* = 0.55) while the second corpus not (Santa Barbara, *p* = 0.07). In contrast, using the method we used, where the parameters are estimated and the fit between the expected and observed frequency distribution is calculated, all three American-English corpora (the child-directed one and the two adult ones) were found to show a very good fit to a Zipfian distribution (**CDS**: alpha = 1.52; beta = 18.34; *r* = 0.965; **Santa Barbara**: alpha = 1.36; beta = 9.23; *r* = 0.99; **CallFriend**: alpha = 1.35, beta = 11.5, *r* = 0.99).

Finally, to further probe the possible inconsistencies generated by this method, we applied the Clauset method to the smaller samples we used to assess alpha for each language (100,000 tokens each, nine languages, see Study 1 in page 18). In Study 1, we used these samples to investigate the stability of our alpha estimations, and to ensure that this corpus size is sufficient to obtain a reliable estimate of alpha. We now wanted to see whether different samples of the same corpus are consistently identified as being drawn (or not drawn) from a power-law distribution. While the estimated alpha was relatively similar (very small SD) across different samples of the same language, the *p*-value using the Clauset method was not (it resulted in different results for different samples from the same language). For example, in British-English 32 samples out of 69 received *p* <= 0.05, and 37 samples received *p* > 0.05. A similar pattern was found for the other languages we looked at (see Table B1).

**Table B1.** Summary of application of Clauset’s method using the poweRlaw package on the full corpora for each language.

**Language**	**Number of sub-samples receiving *p-value > 0.05***	**Number of sub-samples receiving *p-value =< 0.05***	***Measures of alpha across sub-samples* (mean (SD))**
British English	37	32	1.769 (0.064)
Dutch	3	8	1.703 (0.039)
French	15	4	1.748 (0.052)
German	6	15	1.69 (0.067)
Hebrew	3	0	1.937 (0.04)
Japanese	7	7	1.769 (0.084)
Portuguese	2	1	1.717 (0.059)
Spanish	5	1	1.797 (0.075)
Swedish	3	0	1.793 (0.012)

Future work is needed to understand why the method generates such inconclusive results for linguistic data (which is beyond the scope of the current paper). Since our results are not dependent on the distribution being a ‘true’ power law, we did not pursue this method of estimation further.
