# Supplementary material for: Zipfian Distributions in Child-Directed Speech
Source: Open Mind (Camb). 2023 Jan 24;7:1–30. doi: 10.1162/opmi_a_00070 (PMC9987348; doi:10.1162/opmi_a_00070)
Supplement: Supplementary file 1 [file opmi-07-1-s001.pdf]

https://doi.org/10.1162/opmi_a_00070

Supplementary Material

Table S1. Summary of corpora measures across development for Study 2 for German, French, Japanese and Hebrew.

Language	Child's Age Range	No. Tokens	No. Types	Word Frequency (per million)	α	β	Pearson's-r
German	0;7-0;11	988	403	1 - 36 (1012.15 - 36437.25)	-	-	-
	1;0-1;5	96399	5137	1 - 3907 (10.38 - 40529.47)	1.35	8.62	0.995
	1;6-1;11	250243	9070	1 - 8472 (4.0 - 33855.09)	1.4	11.68	0.996
	2;0-2;5	948883	21204	1 - 30903 (1.05 - 32567.77)	1.41	12.23	0.997
	2;6-2;11	652846	19244	1 – 21041 (1.53 - 32229.65)	1.39	11.24	0.998
	3;0-3;5	194773	9681	1 - 6159 (5.13 - 31621.43)	1.36	10.7	0.998
	3;6-3;8	27514	3335	1 - 950 (36.35 - 34527.88)	-	-	-
					Mean=1.38 SD=0.03	Mean=10.89 SD=1.39	Mean=0.997 SD=0.001
French	0;11-0;11	6929	868	1 - 327 (144.32 - 47192.96)	-	-	-
	1;0-1;5	192914	6387	1 - 9222 (5.18 - 47803.68)	1.52	16.53	0.978
	1;6-1;11	327364	9212	1 - 12401 (3.05 - 37881.38)	1.48	15.47	0.994
	2;0-2;5	412244	9600	1 - 15626 (2.43 - 37904.74)	1.51	16.85	0.993
	2;6-2;11	384360	9969	1 - 14417 (2.60 - 37509.11)	1.49	16.9	0.991
	3;0-3;5	193880	7185	1 - 7062 (5.16 - 36424.60)	1.48	16.53	0.991
	3;6-3;11	114253	5527	1 - 4650 (8.75 - 40699.15)	1.44	15.28	0.987
	4;0-4;5	73173	4397	1 - 2711 (13.67 - 37049.18)	1.44	16.12	0.99
	4;7-4;11	22481	2274	1 - 711 (44.48 - 31626.71)	1.38	14.2	0.994
					Mean=1.48 SD=0.03	Mean=16.24 SD=0.64	Mean=0.989 SD=0.005
Japanese	0;6-0;11	2887	484	1 - 190 (346.38 - 65812.26)	-	-	-
	1;0-1;5	66623	5075	1 - 2793 (15.01 - 41922.46)	1.26	7.5	0.995

https://doi.org/10.1162/opmi_a_00070

	1;6-1;11	144204	7178	1 - 7168<br>(6.93 - 49707.36)	1.28	6.7	0.995
	2;0-2;5	214916	9806	1 - 11136<br>(4.65 - 51815.59)	1.26	6.24	0.991
	2;6-2;11	255479	12271	1 - 12210<br>(3.91 - 47792.58)	1.25	5.81	0.997
	3;0-3;5	230124	12373	1 - 11216<br>(4.35 - 48738.94)	1.24	5.9	0.993
	3;6-3;11	198059	11268	1 - 9850<br>(5.05 - 49732.66)	1.23	5.77	0.991
	4;0-4;5	79431	6942	1 - 3595<br>(12.56 - 45259.41)	1.19	5.23	0.994
	4;6-4;11	54473	5773	1 - 2456<br>(18.36 - 45086.56)	1.17	5.06	0.991
					Mean=1.24<br>SD=0.04	Mean=6.03<br>SD=0.79	Mean=0.993<br>SD=0.002
**Hebrew**	1;0-1;5	5703	1349	1 - 266<br>(175.35 - 46642.12)	-	-	-
	1;6-1;11	69794	5140	1 - 3976<br>(14.33 - 56967.65)	1.16	3.06	0.995
	2;0-2;5	119972	7838	1 - 6410<br>(8.34 - 53429.13)	1.16	3.18	0.997
	2;6-2;11	78251	6579	1 - 4130<br>(12.78 - 52778.88)	1.14	3.19	0.997
	3;0-3;5	22701	3367	1 - 1092<br>(44.05 - 48103.61)	-	-	-
	3;6-3;11	8638	1998	1 - 383<br>(115.77 - 44338.97)	-	-	-
					Mean=1.15<br>SD=0.01	Mean=3.14<br>SD=0.07	Mean=0.996<br>SD=0.001

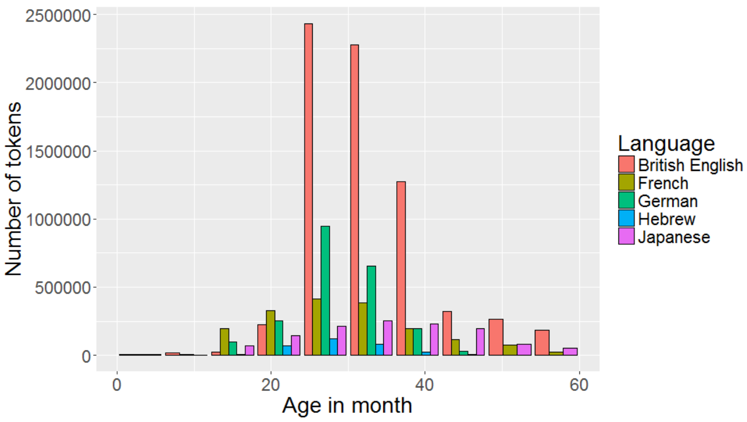

A)

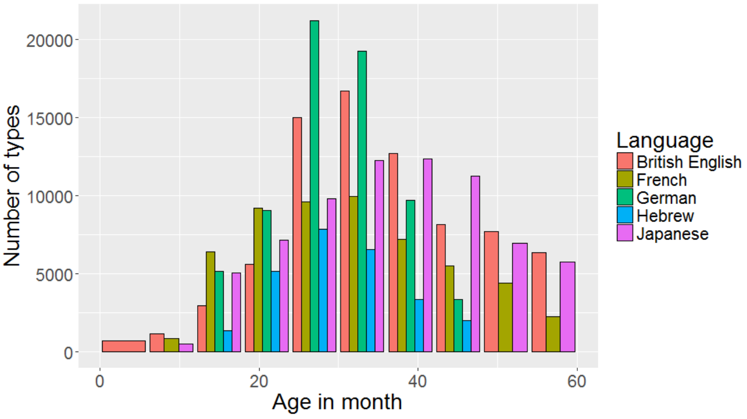

B)

Figure S1. Number of tokens (A) and number of types (B) for each age bin in months, by language.

https://doi.org/10.1162/opmi_a_00070

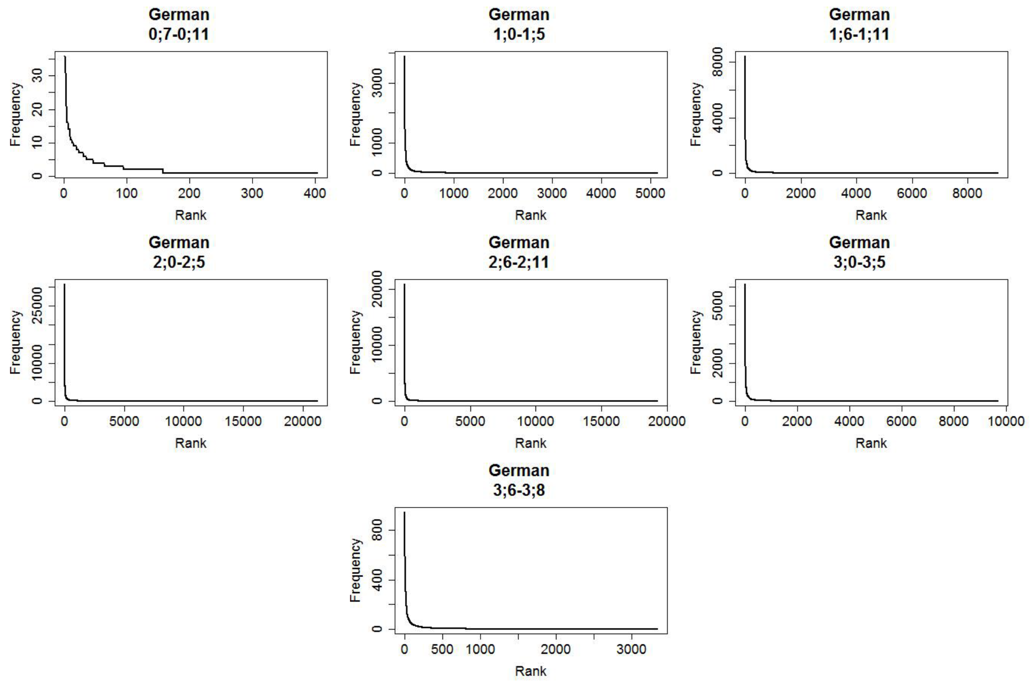

Figure S2. The observed word distribution for all age bins for German. Including age bins that did not reach the minimal size requirement.

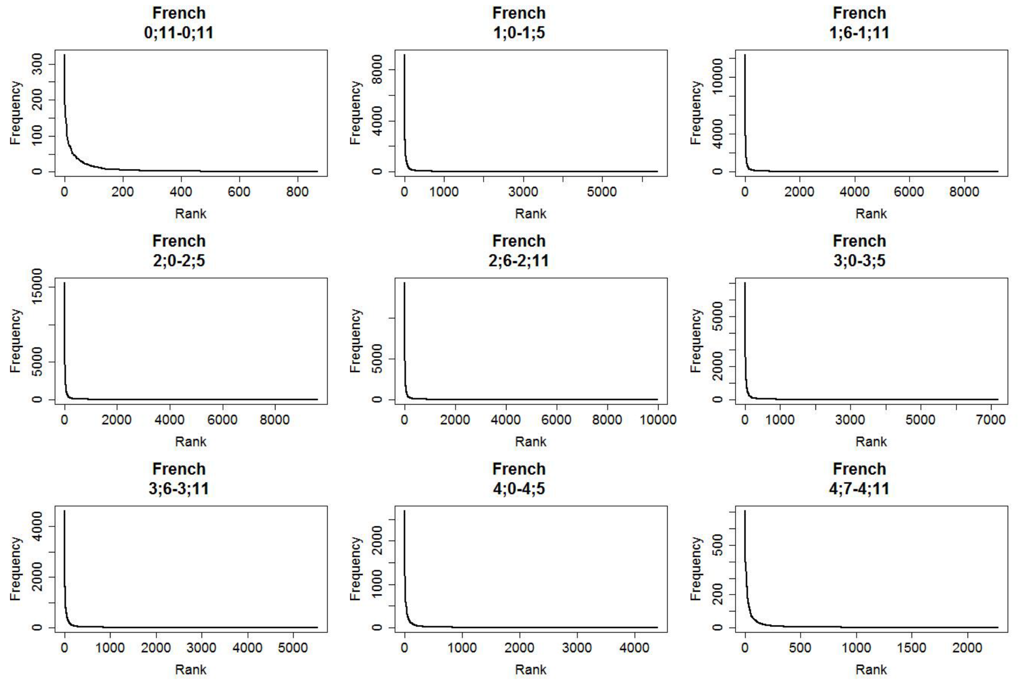

Figure S3. The observed word distribution for all age bins for French. Including age bins that did not reach the minimal size requirement.

https://doi.org/10.1162/opmi_a_00070

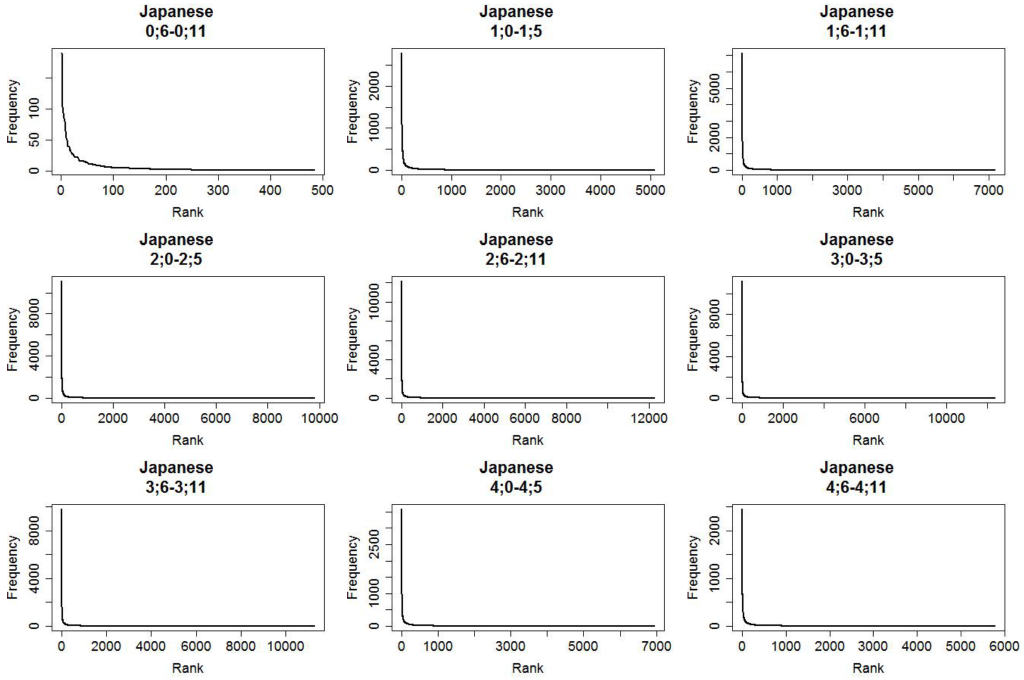

Figure S4. The observed word distribution for all age bins for Japanese. Including age bins that did not reach the minimal size requirement.

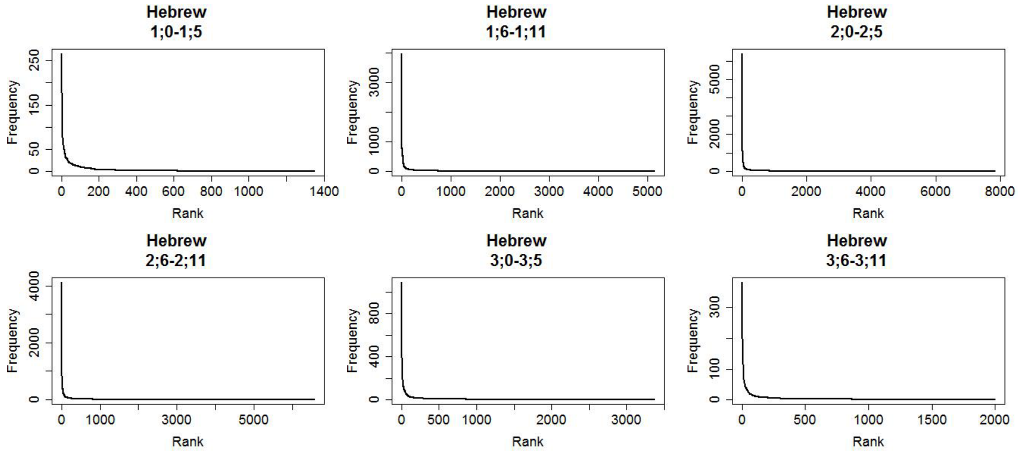

Figure S5. The observed word distribution for all age bins for Hebrew. Including age bins that did not reach the minimal size requirement.

https://doi.org/10.1162/opmi_a_00070

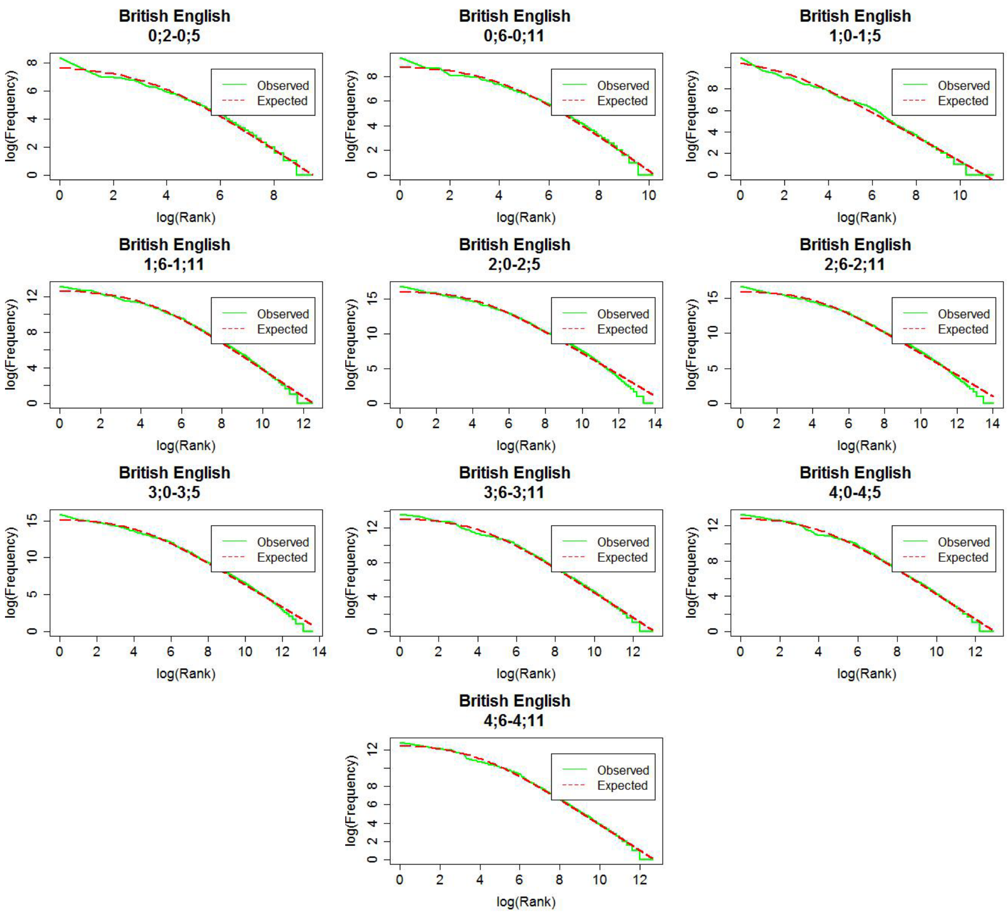

Figure S6. The observed (in green) and expected (in red) distributions on a log-log scale, for all British-English age bins that passed the minimal size requirements

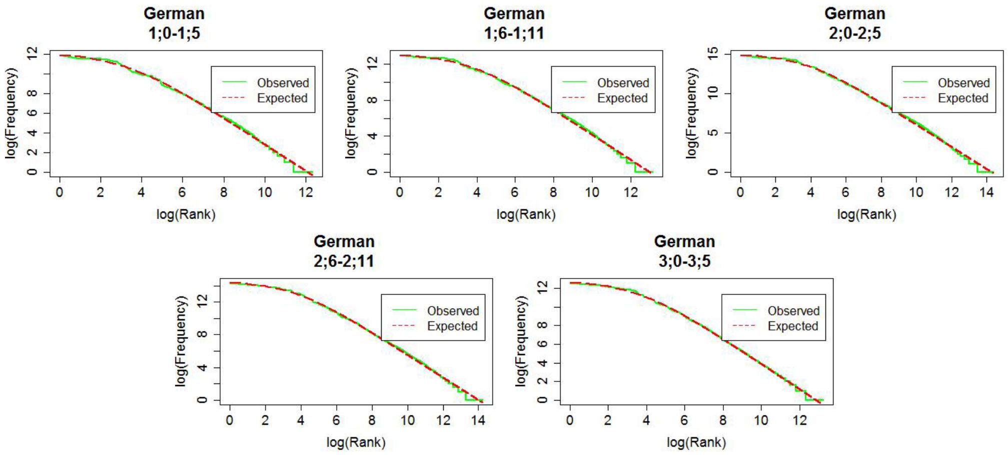

Figure S7. The observed (in green) and expected (in red) distributions on a log-log scale, for all German age bins that passed the minimal size requirements

https://doi.org/10.1162/opmi_a_00070

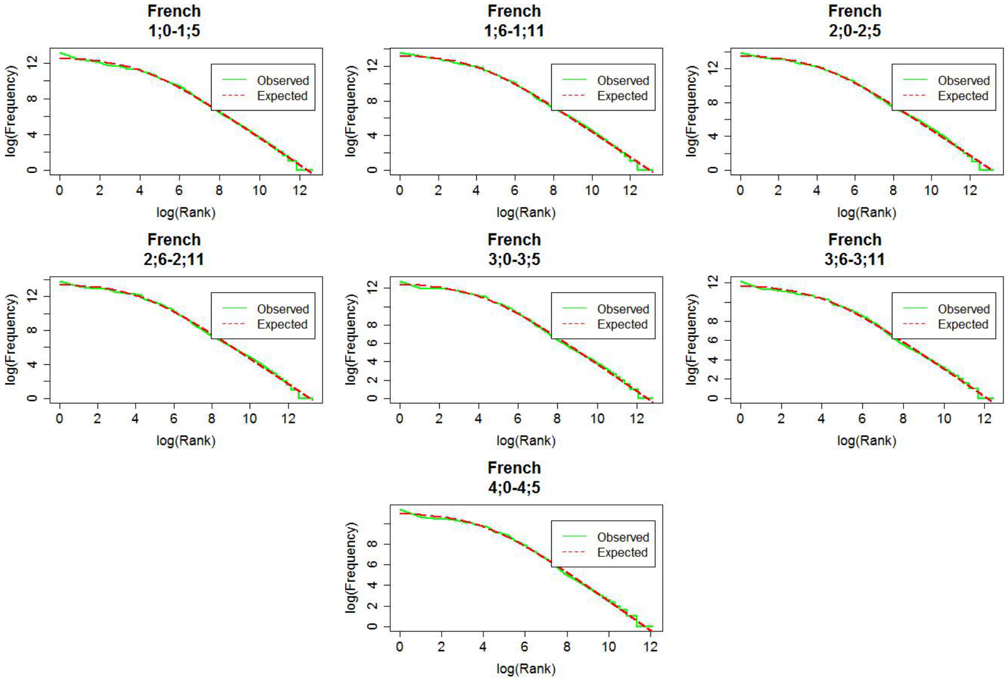

Figure S8. The observed (in green) and expected (in red) distributions on a log-log scale, for all French age bins that passed the minimal size requirements

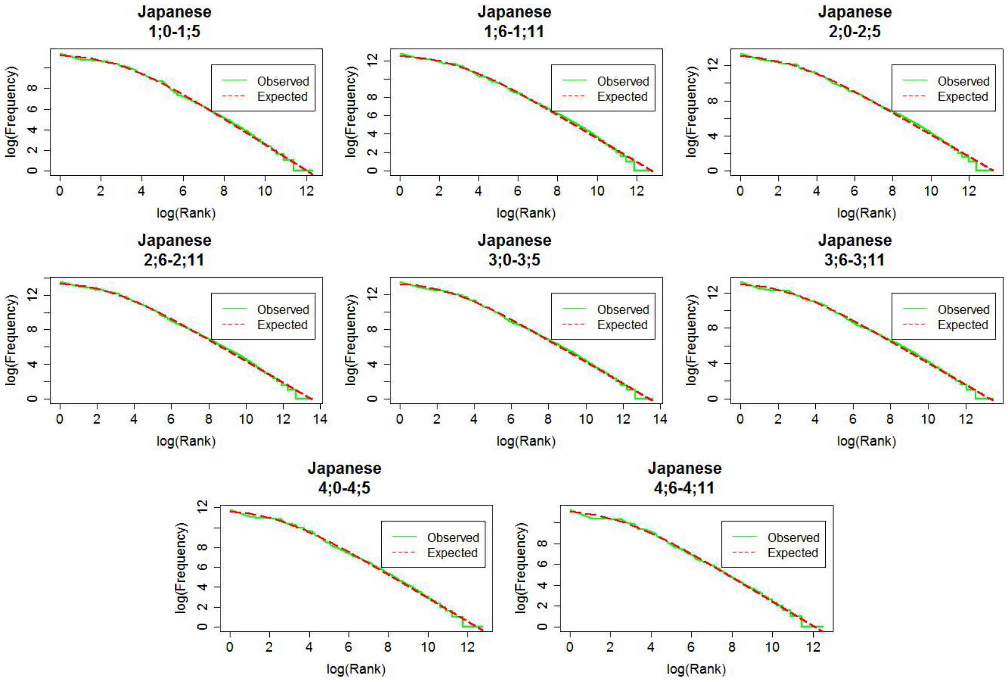

Figure S9. The observed (in green) and expected (in red) distributions on a log-log scale, for all Japanese age bins that passed the minimal size requirements

https://doi.org/10.1162/opmi_a_00070

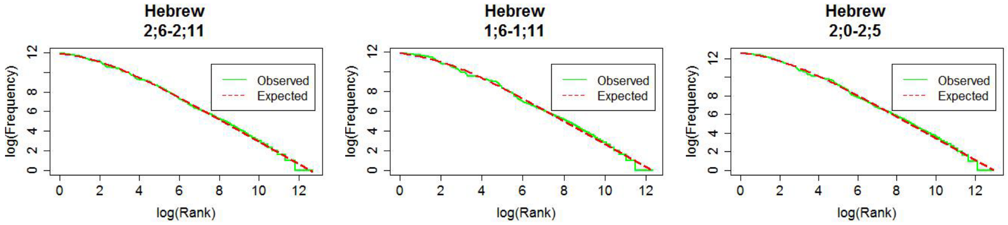

Figure S10. The observed (in green) and expected (in red) distributions on a log-log scale, for all Hebrew age bins that passed the minimal size requirements

https://doi.org/10.1162/opmi_a_00070

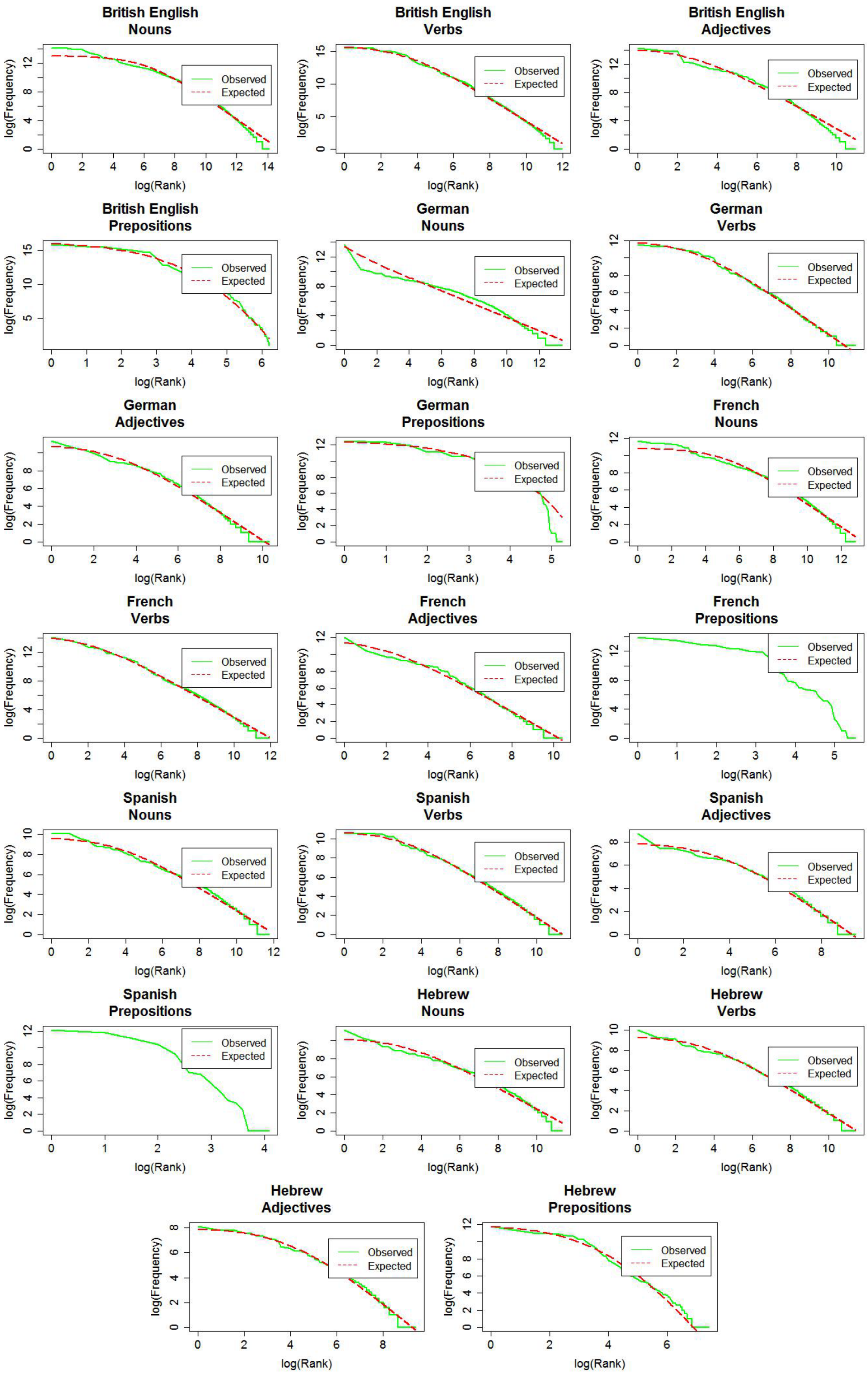

Figure S11. The observed (in green) and expected (in red) distributions for British English, German, French, Spanish and Hebrew for four parts of speech. Results are shown in a log(frequency)-log(rank) coordinates.
